# Assessing the impact of knowledge communication and dissemination strategies targeted at health policy-makers and managers: an overview of systematic reviews

**DOI:** 10.1186/s12961-021-00780-4

**Published:** 2021-12-06

**Authors:** Evelina Chapman, Tomas Pantoja, Tanja Kuchenmüller, Tarang Sharma, Robert F. Terry

**Affiliations:** 1 Oswaldo Cruz Foundation, Brasilia, Brazil; 2 Family Medicine Department, School of Medicine, Pontificia Universidad Catolica de Chile, Santiago, Chile; 3 Evidence to Policy and Impact, Research for Health - Science Division - World Health Organization, Geneva, Switzerland; 4 Evidence to Policy, Copenhagen, Denmark; 5 Manager Research Policy, The Special Programme for Research and Training in Tropical Diseases, World Health Organization, Geneva, Switzerland

**Keywords:** Knowledge translation, Evidence-informed policy-making/makers, Decision-making/makers, Manager

## Abstract

**Background:**

The use of research evidence as an input for health decision-making is a need for most health systems. There are a number of approaches for promoting evidence use at different levels of the health system, but knowledge of their effectiveness is still scarce. The objective of this overview was to evaluate the effectiveness of knowledge communication and dissemination interventions, strategies or approaches targeting policy-makers and health managers.

**Methods:**

This overview of systematic reviews used systematic review methods and was conducted according to a predefined and published protocol. A comprehensive electronic search of 13 databases and a manual search in four websites were conducted. Both published and unpublished reviews in English, Spanish or Portuguese were included. A narrative synthesis was undertaken, and effectiveness statements were developed, informed by the evidence identified.

**Results:**

We included 27 systematic reviews. Three studies included only a communication strategy, while eight only included dissemination strategies, and the remaining 16 included both. None of the selected reviews provided “sufficient evidence” for any of the strategies, while four provided some evidence for three communication and four dissemination strategies. Regarding communication strategies, the use of tailored and targeted messages seemed to successfully lead to changes in the decision-making practices of the target audience. Regarding dissemination strategies, interventions that aimed at improving only the reach of evidence did not have an impact on its use in decisions, while interventions aimed at enhancing users’ ability to use and apply evidence had a positive effect on decision-making processes. Multifaceted dissemination strategies also demonstrated the potential for changing knowledge about evidence but not its implementation in decision-making.

**Conclusions:**

There is limited evidence regarding the effectiveness of interventions targeting health managers and policy-makers, as well as the mechanisms required for achieving impact. More studies are needed that are informed by theoretical frameworks or specific tools and using robust methods, standardized outcome measures and clear descriptions of the interventions. We found that passive communication increased access to evidence but had no effect on uptake. Some evidence indicated that the use of targeted messages, knowledge-brokering and user training was effective in promoting evidence use by managers and policy-makers.

**Supplementary Information:**

The online version contains supplementary material available at 10.1186/s12961-021-00780-4.

## Background

Knowledge translation (KT) seeks to address the challenges involved in the use of research evidence by different and diverse stakeholders, in order to close the gap between the evidence generated and the decisions made by these stakeholders (KT for action). In recent years, KT processes targeted at healthcare providers and patients have been addressed by a number of publications [1–3]. However, such processes targeted at managers or policy-makers have been less researched. Therefore, our overview was aimed at better understanding the impact of KT processes targeted at managers or policy decision-makers.

Despite conceptual differences, the term “knowledge translation” has frequently been used interchangeably with the term “evidence-informed decision-making” [4], as well as other terms such as “knowledge transfer”, “knowledge exchange”, “research utilization” and “implementation” [5]. KT has also been conceptualized as a term to describe the range of strategies to address the barriers to evidence-informed decision-making [6]. According to WHO, KT is defined as “the exchange, synthesis, and effective communication of reliable and relevant research results. The focus is on promoting interaction among the producers and users of research, removing the barriers to research use, and tailoring information to different target audiences so that effective interventions are used more widely” [7].

In this context, diffusion, dissemination and implementation have been described in research findings by identifying and overcoming barriers through specific multifaceted interventions (“make it happen”) [8, 9] along a continuum of intensity for KT activities designed to promote the use of research evidence in decision-making processes [8]. Diffusion or communication activities are those that are passive and largely unplanned, uncontrolled, and primarily horizontal or mediated by peers (“let it happen”).

Dissemination focuses primarily on communicating research results by targeting and tailoring the findings and the message to a particular target audience (“helping it happen”). Finally, in this taxonomy, implementation involves systematic efforts to encourage the adoption of the research findings by identifying and overcoming barriers through specific multifaceted interventions (“make it happen”) [8, 9].

For the purposes of this review, we focused on communication and dissemination interventions/strategies/approaches (although these terms are used for different purposes in the literature, we will use them interchangeably to be inclusive) targeted towards policy-makers and health managers. As mentioned before, dissemination refers to identifying the appropriate audience and tailoring, targeting or framing the message to that audience. This could include different research products such as evidence-based guidelines, publications, research reports, pamphlets, videos and websites, disseminated through different interventions/strategies/approaches depending on the target group. Furthermore, conceptual frameworks underlying each intervention/strategy/approach and tools used in its implementation could be identified.

### Review question

What is the effectiveness/impact of the different knowledge communication and dissemination interventions/strategies/approaches targeted to health policy-makers and managers?

## Methods

This overview of systematic reviews (SRs) adhered to the Preferred Reporting Items for Systematic Reviews and Meta-Analyses (PRISMA) statement [10]. An SR protocol was developed and registered in PROSPERO (CRD42021237214) prior to undertaking the searches.

### Inclusion criteria for studies

Studies were selected based on the following inclusion criteria:

#### Types of studies

SRs including primary studies of any design providing information on the effectiveness of dissemination strategies were included. When no SRs were identified for a specific intervention/strategy, a focused search for primary studies in PubMed was conducted.

As no relevant primary studies were located , SRs focused on a different audience (health professionals or recipients of care) were assessed as indirect evidence, which means this study remained focused on SRs only.

#### Types of participants

Studies including only managers or policy-makers as the target audience or studies including a larger group (e.g. also healthcare providers and patients) but in which data for different target audiences was reported separately were included. Because the definitions of managers and policy-makers are not always precise, we used a broad definition including all types of decision-makers such as hospital directors or administrators, health administrators, department chiefs, health planners, and programme directors or managers.

Intervention(s), exposure(s): The definitions of KT communication and dissemination strategies were adapted from the classification proposed by McCormack et al. [11] and are described in greater detail in Additional file 4: Tables S2 and S3:

- Techniques to communicate evidence through (1) tailoring the message, (2) targeting the message, (3) using narratives, (4) framing the message and (5) using a multicomponent approach.
- Strategies for disseminating evidence to (1) increase the reach of the evidence, (2) increase people’s motivation to use and apply the evidence, (3) increase people’s ability to use and apply the evidence, and (4) use a multipronged approach with any of the dissemination strategies described above.

The communication or dissemination support could be physical materials (i.e. pamphlets, flyers, board games, policy briefs), electronic (i.e. video, audio-drama), Internet-based (i.e. databases, information services, discussion lists, registries of preprocessed research evidence or online-tailored and targeted messaging, online training, range of epidemiological and demographic data to inform public health policy and programme decisions, etc.), interpersonal (workshops, knowledge brokers, dialogues), through media (radio, TV) or social media (Twitter, Facebook, YouTube, including blogs and forums, etc.), and others (i.e. visual arts, literary arts, performing arts and applied arts).

The strategies could be defined as single or combined. The combined or multicomponent strategies refer to different combinations of single strategies—targeted messages + knowledge brokers; education + information service + free access to databases; small group workshops + one-to-one consultations, etc.

### Comparator(s)/control

We did not have restrictions on types of comparisons.

### Types of outcome measures

We included outcomes related to the effectiveness of communication and dissemination strategies targeted at managers or policy-makers.

### Primary outcomes

Our primary outcomes were use or uptake of research results, decision-making, adherence to research knowledge (i.e. change in knowledge/awareness) and behavioural change.

### Secondary outcomes

Secondary outcomes were those related to understanding, perception and persuasiveness. We considered only objective understanding and not self-reported understanding. Perception referred to how effective an intervention was perceived to be. Persuasiveness considered how likely participants were to make a hypothetical decision in favour of an intervention. Costs and cost-effectiveness of strategies were also considered when data were available. Additionally, we considered barriers to uptake of research evidence through communication or dissemination strategies, underlying theories behind the strategies and research gaps.

### Search methods for identification of studies

A comprehensive electronic search of 13 databases and a manual search in four websites was conducted. The databases searched were MEDLINE (PubMed), EMBASE, Health Systems Evidence, the Cochrane Library, CINAHL (EBSCOhost), PsycINFO (EBSCOhost), the Campbell Collaboration, EPPI-Centre database of health promotion research (BiblioMap), WorldWide Science, Education Resources Information Center (ERIC), DoPHER [database of promoting health effectiveness reviews], Epistemonikos and PDQ-Evidence. The manual search was carried out in Google including Google Scholar, Rx for Change, International Initiative for Impact Evaluation (3ie) and Institute of Education Sciences (IES). The reference lists of included studies were also searched to identify other relevant literature not picked up by our search.

## Search strategy

Searches were conducted between 30 January and 4 February 2021, and supplementary searches (reference lists) were performed until 24 February 2021. Databases were searched using keywords from both keyword areas—combined using “AND”. Keywords were searched for in the title and abstract fields. Results were downloaded into the EndNote reference management programme (version X9) and duplicates removed. The internet search utilized the search terms: “policy-makers” OR policymak* OR manager; “research utilization” OR “research communication” OR “information dissemination”; “knowledge translation”; “research use”; and “research uptake”; combined with “systematic review”.

Search terms and strategies for the electronic searches are presented in Additional file 1.

SRs in English, Spanish or Portuguese (because the reviewers are familiar with these languages) during the past 15 years were included. We included both published and grey literature.

### Screening and selection of studies

Searches were conducted and titles and abstracts were screened independently by two review authors (EC and TP) according to the selection criteria. The full texts of any potentially relevant papers were retrieved for closer examination. The inclusion criteria were then applied against the full-text version of the papers independently by two reviewers (EC and TP).

Disagreements regarding the eligibility of studies were resolved by discussion and consensus. All studies which initially appeared to meet the inclusion criteria but on inspection of the full-text paper did not meet the inclusion criteria are listed in Additional file 2, together with reasons for their exclusion.

### Data extraction

Information extracted from the included studies considered the type of synthesis document (SR, scoping review or rapid review, etc.), objectives, study design and number of studies included, date of last search, participants (type of decision-maker), settings, country of study, intervention/strategy and domain (communication, diffusion or dissemination), outcome measures, findings, barriers, research gaps, underlying theory and financing source. We limited data extraction to SRs assessing the effectiveness of strategies targeted at policy-makers or managers.

Data were extracted by two reviewers (EC and TP). Each reviewer extracted 50% of the included studies, and then 40% of the extractions were cross-checked. Doubts or disagreements were resolved through discussion and consensus. During the process of extracting data from the included SRs, reviewers completed a matrix containing the different types of interventions and the outcomes measured in each included SR.

Additionally, we disaggregated the formats or tools used to facilitate or support the communication and dissemination strategies. Classification was done by two reviewers (EC and TP).

### Assessment of methodological quality

The methodological quality of the included SRs was evaluated using AMSTAR 2 (A MeaSurement Tool to Assess systematic Reviews 2) [12]. Scoring was done by two reviewers (EC and TP); each evaluated 50% of the included studies, of which 40% were then cross-checked. Doubts or disagreements were resolved through discussion and consensus.

### Data analysis

Findings from the SRs were synthesized using tables and a narrative summary. Meta-analysis was not possible because the included studies were too heterogeneous in terms of the populations, strategies/interventions tested, and outcomes measured. Few SRs informed effectiveness measures; therefore, to inform the main results, we developed effectiveness statements using four categories and standardized language as proposed by Ryan et al. [13, 14]. The decision rules considered the results, their statistical significance, and the quality and number of studies that supported the results. The four categories were (1) sufficient evidence, (2) some evidence, (3) insufficient evidence and (4) insufficient evidence to determine effectiveness (more detailed definitions of each category are provided in Additional file 4). Category 4 was also used to complement research gaps.

## Results

### Search results

**Fig. 1:**
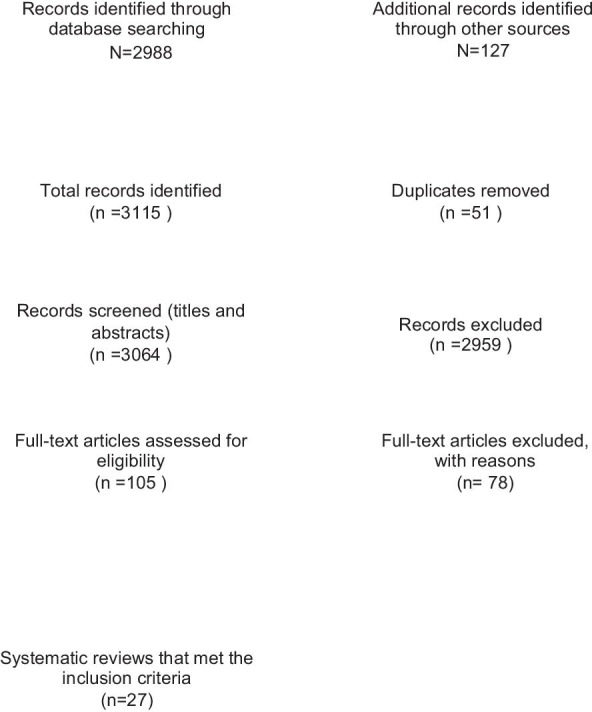
Study selection flow chart

Twenty-seven SRs met the inclusion criteria for the overview [6, 15–40]. The selection process for SRs and the number of papers found at each stage are shown in Fig. 1. The reasons for the exclusion of 78 papers at full-text stage are shown in Additional file 2.

### Characteristics of included studies and quality assessment

Details of the characteristics of the included SRs (we also synonymously use “studies” interchangeably when we report our results) and AMSTAR 2 scores are presented in Additional file 3 and Additional file 5. Of the 27 SRs, two were reported as rapid reviews [33, 40] and two as scoping reviews [29, 38].

Regarding AMSTAR 2, six SRs had high ratings [15–19, 23], one had a moderate rating [6], six had low ratings [20–22, 24–26] and 14 had critically low ratings [27–38].

Of the 27 SRs, two only included randomized controlled trials [21, 23], six included both experimental and quasi-experimental designs [15, 17, 19, 25, 30, 32], and 19 included both quantitative and qualitative research [6, 16, 18, 20, 22, 24, 26–40]. Thirteen SRs included only policy-makers or managers as the target [6, 18, 22, 25–27, 31–34, 37–39], and the remaining 14 also included healthcare workers, researchers, legislative staff members, the public, patients, students and other stakeholders such as nongovernmental organizations or international organizations.

Four reviews included only low-income countries [22, 28, 36, 37], 12 included only high-income countries [6, 20, 21, 25, 26, 30–34, 39, 40], one did not report [15], and the remaining 10 included low–medium- and high-income countries.

Twelve SRs mentioned or were informed by a theory or framework [15, 16, 18–20, 28, 29, 31, 34, 35, 39, 40], none of which was used by more than one SR. Some examples of the theories used were the Rothman and Salovey theory, arts-based KT (ABKT) framework, the Guiding Arts-Based Research Assessment framework (GABRA), knowledge-brokering domains, policy framework (Shiffman and Smith framework), leadership theory, domains of knowledge management (Lee et al. 2010), Kirkpatrick’s evaluation model hierarchy, theory-driven approach, Green’s transportation imagery model, and the SPIRIT (Supporting Policy In health with Research: an Intervention Trial) action framework. More detail can be found in Additional file 8.

### Types of communication or dissemination strategies

Three studies included only a communication strategy [15, 16, 23], eight studies included only dissemination strategies [24, 26, 29, 31, 32, 35–37], and the remaining 16 included both. Many reviews included more than one communication or dissemination strategy using different formats (the latter were also called tools) such as electronic or internet-based, to facilitate outreach. For more detail see the Additional file 6: Tables S1–S3.

Regarding strategies to communicate evidence, we found 12 SRs reporting “Targeting the message”, 11 reporting “Tailoring the message”, four reporting a “Multicomponent approach”, three reporting “Using narratives and other forms of art”, and three reporting “Framing the message”. There was just one study reporting “Using different presentation formats”.

Regarding strategies to disseminate evidence, we found the following: “Increase people’s motivation to use and apply the evidence” 18 studies, “Increase reach of the evidence” studies, “Increase people’s ability to use and apply the evidence” 12 studies, and for the “Use a multipronged approach” six studies.

### Different formats or tools to facilitate or support the communication and dissemination strategies

Eight studies used “physical or printed tools”, for example, drawing, painting, research summaries, journal prints and bulletins; 13 used an “electronic tool”, such as simulation games, multimedia, television appearances, entertainment education (prime-time network TV storyline), short films, videos, PowerPoint presentations and handouts. Seventeen studies reported “tools based on Internet”. Highlighted mong these were specific websites (e.g. Health-Canada), access to a repository of SRs, web-based training programmes, structured seminar series, networks, access to research and to databases, web-based information and communication, dissemination of SRs through the website, and series of emails with links to full references, abstracts and summaries. Regarding “interpersonal” communication or dissemination skills—understood as any interaction between two or more people face-to-face or not (such as using telephone or video calls or otherwise)—we found 20 studies reporting targeted messaging and knowledge brokers, engagement with arts-based projects including elements of training or skills-building, knowledge-brokering (the most frequently mentioned), small group workshops, one-to-one consultations, collaborative approaches, structured seminar series to promote interaction between policy-makers and researchers, conference technology to support knowledge sharing, networks to support knowledge production and exchange, organization-wide capacity-development initiatives, grant-funded collaboration involving policy-makers, deliberative dialogues, interactions and interpersonal connections with staff, tailored exchanges within and across departments and disciplines, face-to-face exchange, KT platforms (KTPs), and research engagement actions such as increasing the interaction between decision-makers and researchers. Three studies reported the use of “media or social media”. They included, among others, media and public opinion, television appearances, entertainment education (prime-time network TV storyline), short films, magazines and journal prints, narrative action reflection workshops, conference technology to support knowledge sharing, communities of practice, booklets with testimonies, advocacy summaries of personal experiences, wikis, blogs and online forums. For more details see Additional file 6: Tables S2 and S3.

### Types of outcomes

Many reviews included data for more than one primary or secondary outcome; therefore, the number of outcomes measured may exceed the number of included SRs. For greater detail see Additional file 7.

#### Primary outcomes

Thirteen studies assessed the use or uptake of research results, 14 studies assessed decision-making or changing behaviours, six studies assessed intention to use or apply evidence, 14 studies assessed change in knowledge, and five studies assessed changes in awareness.

#### Secondary outcomes

Understanding was assessed by nine studies, perception by seven studies and persuasiveness by three studies, and cost was reported by a single study as a research gap.

Additionally, the included studies assessed outcome measures that were not included in our protocol. These included learning (six studies), attitudes/beliefs (four studies), skills or competencies (three studies), discussion regarding the evidence (two studies), health outcomes (two studies), engagement (two studies), policy changes (one study), value of research evidence (one study), scaling-up of intervention (one study), acceptability (one study), research culture (one study), intention to act (one study), sustainability of evidence-informed policy-making (EIPM) (one study), research coproduction (one study) and credibility (one study). More details are provided in Additional file 7: Table S1.

### Effectiveness statements

The effects of interventions are presented below by each strategy according to the adopted taxonomy [13]. Some reviews evaluated multiple interventions and so contributed evidence to more than one category. None of the selected SRs provided “sufficient evidence” (category 1 in the evidence rating scheme used, detailed in Additional file 4: Table S1) for any of the strategies. In Tables 1 and 2, we present the effectiveness statements with “some” or “insufficient evidence” (categories 2 and 3 in Additional file 4: Table S1) for communication and dissemination strategies, respectively. The findings rated as having “insufficient evidence to determine” (category 4) were used to inform the research gaps. More details are provided in Additional file 7: Tables S2 and S3.

**Table 1:** Effectiveness statements for the communication strategies (categories 2 and 3)

Some evidence	Insufficient evidence
Tailoring the message	
“An intervention which combined access to relevant systematic reviews with tailored and targeted messages led to changes in public health practice/decision-making” [30]	The studies included in this review provide some evidence that the use of tailored targeted messages, with access to registries of research, may increase the use of research in policy development [32] (citing Dobbins 2009) “…the tailored message intervention group was associated with a significant increase in the use of evidence in recent public health policies and programs (*p* < 0.001)” [33] “Three studies of low-to-moderate risk of bias, identified interventions that showed a statistically significant improvement: educational visits, short summaries of systematic reviews, and tailored and targeted messaging” [19]
Targeting the message	
“An intervention which combined access to relevant systematic reviews with tailored and targeted messages led to changes in public health practice/decision-making” [30]	“Targeted messaging (was) significantly more effective in promoting EIDM [evidence-informed decision-making] than other strategies (*p* < 0.009) [27] “Tailored targeted messages reportedly improved level 3 behaviour change outcomes” [18]
Using narratives	
None identified	“The evidence base on the effectiveness of arts-based approaches in engaging the public (or policy-makers) in research is limited by the lack of systematic evaluation” [40]
Framing the message	
Direct evidence: None identified Indirect evidence: “When attributes of health information are framed negatively (e.g., chance of mortality with cancer) understanding may be better than when the same information is framed positively (e.g., chance of survival with cancer). However, perception may be better when it is positively framed” [15] “When goals of health information are framed as loss messages (e.g., ‘if you do not undergo screening test for cancer, your survival will be shortened’) there may be a more positive perception of effectiveness for screening messages and may be more persuasive for treatment messages than when framed as gain messages” [15]	None identified as direct evidence
Using different presentation formats	
“Two studies assessed the use of evidence summaries (compared with complete systematic reviews) in decision-making and found little to no difference in effect. There was also little to no difference in effect for knowledge, understanding or beliefs (four studies), and perceived usefulness or usability (three studies). Summary of findings tables and graded entry summaries were perceived as slightly easier to understand compared to complete systematic reviews. Two studies assessed formatting changes and found that for summary of findings tables, certain elements, such as reporting study event rates and absolute differences, were preferred as well as avoiding the use of footnotes” [23]	None identified as direct evidence
Multicomponent communication techniques	
None identified	“All the studies employed at least three strategies to increase the use of evidence, mainly with regards to the implementation of a particular evidence-based policy. No included study allowed us to estimate the effectiveness of individual strategies to increase the use of evidence” [39]

**Table 2:** Effectiveness statements for the dissemination strategies (categories 2 and 3)

Some evidence	Insufficient evidence
Increase reach	
“Simply having access to an online registry of research evidence appeared to have no impact on evidence-informed decision making” (Dobbins 2009 cited in [30])	“…the extent to which these (knowledge-translation) resources are used and are found useful by policymakers is unclear” [38] “Promising interventions include … an e-registry of reviews but these interventions need to be developed further” [19]
Increase people’s motivation	
“While knowledge brokering did not have a significant effect generally, results suggested that it did have a positive effect [in terms of research uptake] on those organizations that at baseline perceived their organization to place little value on evidence-informed decision making” [30]	“…knowledge brokering (KB) was more effective in those organizations that placed less value on research evidence and was less effective in those organizations that already recognized the importance of evidence-based decision making” [27] “Only one cluster RCT [randomized controlled trial] evaluated an organisational intervention (which included a knowledge broker, access to a repository of systematic reviews and provision of tailored messages), and reported no statistically significant difference in evidence informed programme planning(mean change − 0.42; 95% CI − 1.10 to 0.26, *P *< 0.45) neither of having access to a knowledge broker (mean change − 0.09; 95% CI − 0.78 to 0.60) [24]
Increase people’s ability to use	
“Tailored interactive workshops supported by goal-focused mentoring, and genuine collaboration, seem particularly promising” [29]	“Training in the appraisal of research and its use appears to increase participants’ skills in critical appraisal and possibly their perceptions about the value of research, but not their use of research” [32] “The findings of pre–post survey data suggest that a 1 day workshop training event for policy-makers and researchers may improve knowledge and understanding of key topics related to partnership research, evidence-informed policy-making and may enhance policy-makers’ research capacity” [35]
Multifaceted dissemination strategy	
Multifaceted KT strategies led to changes in knowledge but not practice [30] “The findings suggest that although a digital TEKT [technology-enhanced knowledge translation] intervention may improve knowledge, the effects of such interventions on other outcomes are equivocal” [21]. (Digital TEKT could make use of social media, email, internet, electronic databases, electronic prompts or reminders, web-based webinars, or training or interactive websites to facilitate research use by end users)	“…the multi-faceted ARC (Availability, Responsiveness and Continuity) organizational approach, which includes a focus on the social context of organizations and the social process of adopting innovations (which are linked to stimulate community support, stimulate better communication and relationships between stakeholders, increase the measure the organization and staff value research evidence, increase the extent to which staff have the knowledge and skills to use the evidence, and increase the extent to which organizations have the tools and systems necessary to support the participation and use of research) can be of great benefit in improving the use of evidence and evidence-based practices” [39]

### Communication strategies

Fourteen reviews assessed the effects of a communication strategy [6, 16–19, 23, 25, 27, 28, 30, 32, 33, 39, 40], but only two [23, 30] provided evidence considered useful for the development of effectiveness statements (Table 1). Nine reviews provided some effectiveness information, which was insufficient, however, to make any definitive statements, and three reviews informed research gaps [16, 28, 33]. The 11 reviews providing evidence of effectiveness assessed interventions focused on tailoring and targeting the message communicated to decision-makers, the use of arts-based approaches to communicate research, the use of alternative formats for presenting SRs, and multicomponent communication strategies. The use of tailored and targeted messages (both strategies are usually implemented together) based on reliable evidence seems to lead to changes not only in knowledge but also in decision-making practices of the target audience [17, 27, 30, 32].

There was little to no difference in the effect of evidence summaries compared with complete SRs on the use, understanding, belief or perceived usefulness of evidence. However, summary of findings tables and graded entry summaries were perceived as slightly easier to understand than complete SRs [23]. The evidence regarding arts-based interventions and multicomponent communication strategies was insufficient to make definitive judgements about their effectiveness [40].

One additional review targeted at healthcare providers and recipients of care provided indirect evidence regarding the effects of different ways of framing the message on the use of evidence [15]. The negative framing of health information showed a better understanding but a worse perception of its attributes compared with a positive framing. Likewise, when goals of health information are framed as loss messages, there seems to be a more positive perception of them and they seems to be more persuasive than when they are framed as gain messages [15].

### Dissemination strategies

Twenty-four reviews assessed the effects of a dissemination strategy [6, 17–40], but only three [21, 29, 30] provided evidence that was useful for the development of effectiveness statements (Table 2).

Thirteen reviews provided some information, but it was insufficient for making definitive effectiveness statements, and the other eight reviews were used to inform research gaps. Interventions aimed mainly at improving the reach of evidence did not seem to have a significant impact on the use of evidence in decision-making processes. On the other hand, interventions designed to motivate target audiences to use and apply evidence (e.g. knowledge brokering) or to enhance recipients’ ability to use and apply evidence (e.g. training workshops with an interactive component) may have a positive effect on decision-making processes in some organizations, but the evidence was inconsistent. Finally, multifaceted dissemination strategies could lead to changes in knowledge about evidence but not in its use in decision-making (especially promising in this group are digital technology-enhanced KT interventions).

### Barriers to communication and dissemination processes

Nine studies reported barriers to the communication and dissemination processes [15, 18, 19, 22, 27, 28, 31, 32, 40]. At the individual level, the lack of time to be trained [18, 22, 27, 31] or to critically read articles [22, 27], and the lack of awareness, familiarity and perceived usefulness of research and motivation [19] were mentioned. At the organizational level, the lack of financial or adequate infrastructure [22, 28, 40], frequent staff turnover [22, 27], the health system in general [15], the lack of access to evidence [19, 22, 27, 32], the lack of leadership [28], an organizational culture [19, 22, 31, 40] not conducive to communication and dissemination processes, and difficulty in establishing alliances [22, 27] or developing a shared vision [18] were identified. Scarce training of human resources and peer support was a specific barrier mentioned with regard to human resources [19, 22].

Finally, barriers related to research itself included the type of research being considered, the need to refine methodologies used for conducting reviews, the need to consider diverse contexts, the perceived credibility of findings, timeliness of research and the relevance of research in day-to-day decision-making [22, 27, 40]. Facilitators related to the format and content of synthesis documents, for instance the inclusion of a format with graded access and executive summary, were mentioned by one review as being helpful [19].

### Research gaps

Eighteen studies reported research gaps [6, 17–40]. The need for more robust studies, especially experimental studies measuring the effectiveness of different communication and dissemination strategies [6, 18, 19, 31, 32, 39] and programmes [25] in different settings [18, 21, 22], including hypothetical scenarios [15], were featured in many of them. Others reported the need to evaluate specific strategies such as the use of knowledge brokers [20], KTPs [24], different forms of narratives or art [16, 40]. One study reported the need for cost-effectiveness studies in KT strategies [28].

Also highlighted was the need for qualitative studies to better understand the knowledge, beliefs and attitudes of policy-makers towards narratives [16] or their attitudes towards research in general [39], user needs [38], and individual attributes and contextual factors that could influence the effectiveness, for example, of knowledge brokers [20].

The description of the interventions was also noted as a gap—for example, the need to include the frequency and duration of the intervention, its components [16, 32], the comparison of different formats [16, 17], the interaction between researchers and decision-makers [32], the interventions in different contexts [16, 22] and the characteristics of the decision-makers themselves as modellers of the interventions [16].

The need for valid and standardized measurements of results and impacts of strategies on policies was highlighted [6, 20–22, 32], as well as the need to incorporate the perceptions of decision-makers [16].

Limited descriptions of frameworks or theories that guide the development of the intervention [6, 16] were also reported.

## Discussion

There is no doubt about the relevance of the use of evidence in the formulation, implementation and evaluation of policies and the growing scientific production in health policy research [41].

We identified 27 SRs, but only four [21, 23, 29, 30] provided some evidence to enable us to develop effectiveness statements for three communication and four dissemination strategies. Additionally, 13 other reviews [6, 17–20, 25, 27, 32, 34, 35, 38–40] provided evidence of effectiveness but it was insufficient to develop definitive statements.

The other nine reviews only provided evidence to inform research gaps. An additional review [15] provided indirect evidence about one communication strategy (different framing of the messages), for which we were unable to find direct evidence targeted to managers or policy-makers. Regarding communication strategies, the use of tailored and targeted messages (both strategies are usually implemented together) based on reliable evidence seems to lead to changes not only in knowledge but also in decision-making practices of the target audience.

Surprisingly, although evidence summaries (graded-entry formats, policy briefs) were perceived as easy to understand, they did not have a relevant effect on the understanding, knowledge, beliefs, perceived usefulness or use of evidence in decision-making. However, this finding was only based on two studies comparing different types of evidence summaries with complete SRs; therefore, more research is needed to determine whether evidence summaries are in fact effective, and also what formats and types of evidence summaries (in terms of length, language for textual or visual, podcasts or other media options, etc.) are optimal for decision-makers. Regarding dissemination strategies, those interventions aimed mainly at improving the reach of evidence do not seem to have a significant impact on the use of evidence in decision-making processes. However, interventions designed to motivate target audiences to use and apply evidence (e.g. knowledge-brokering) or to enhance their ability to use and apply evidence (e.g. training workshops with an interactive component) could have a positive effect on decision-making processes in some organizations, although the evidence was inconsistent. Multifaceted dissemination strategies (e.g. combining improving access to evidence + workshops or multiple technology-enhanced interventions) could lead to changes in knowledge about evidence but not in its use in decision-making. SRs, evidence briefs and other synthesis documents have been established as a powerful tool for policy-makers and managers [41–45]. However, they are not sufficiently available, and specific strategies under the “knowledge translation” label should be used to promote the use of such evidence in their decision-making processes [9]. Knowledge communication and dissemination strategies are a subgroup of KT interventions focused mainly on the “push” of research findings to the target audience. In this sense, communication and dissemination focus primarily on communicating research results by targeting and tailoring the findings and the message to a particular target audience [8]. To our knowledge, this is the first overview of SRs that has attempted to assess the impact of knowledge communication and dissemination strategies targeted exclusively to health policy-makers and managers.

Recent research also suggests some aspects to consider in the decision-makers themselves. A recent study points out that managers should demonstrate basic competencies consisting of knowledge, skills and attitudes necessary to perform managerial tasks and also proposes new competencies that include evidence-informed decision-making, where managers should know how to evaluate and apply the evidence [46]. Something similar has also arisen recently for policy-makers regarding the professional development process in the field of public administration, establishing the skills that a good policy-maker must have, in which the knowledge and use of evidence plays a fundamental role [47].

Regarding the use of theories, frameworks or models to inform the communication or dissemination processes as KT strategies, we found 13 SRs that used or mentioned one or more different frameworks. We assume that such variability depends on the context, key components, objectives of KT, primary target audience, levels of use or characteristics of the users. This variability and high number of frameworks was also reported in various studies as creating confusion for users regarding which to select [48]. While consensus seems to exist regarding the need for using theories, frameworks or models when designing and implementing KT interventions, there is no agreement on a specific framework [48–50].

### How our findings compare with what others have found

In contrast to the substantial evidence available on the effectiveness of communication and dissemination strategies targeting healthcare professionals and consumers [1–3], the body of evidence regarding the effects of strategies targeted at health managers and policy-makers is scarce. Although we were able to identify 27 SRs, only four provided some evidence to enable us to develop effectiveness statements. Our findings, to some extent, mirrored what has been found regarding KT strategies targeted at consumers, where tailored interventions aimed at the acquisition of skills and competencies (increased ability to use evidence in our taxonomy) seem to be more effective, and single interventions that only provide information may improve knowledge, but their effect on decision-making processes is uncertain [3]. Improving communication between healthcare providers and patients and involving patients in the decision-making process were also effective interventions, such as those aimed at increasing people’s motivation to use evidence (such as knowledge brokers) in our classification. Although the range of effective interventions targeted at healthcare professionals is broader than that represented in our findings [1], strategies aimed at increasing the motivation (e.g. educational outreach) and ability to use evidence (e.g. training workshops) were also effective for that target audience. However, more passive interventions such as the distribution of printed educational materials (increasing the reach of evidence) were also effective for healthcare providers, in contrast with the lack of effects that we found.

This could be related to differences in complexity between clinical and managerial/policy decision-making environments, with a more prominent role for evidence in the former and a greater influence of other factors (such as previous decisions, political context) in the latter making less intensive intervention—focused only on the access to evidence—more effective in clinical environments.

Our findings also mirrored, to some extent, those from a similar review of the efficacy of interventions applied to increase decision-makers’ use of research in various decision arenas [51]. Although that study used a taxonomy of interventions based on the underlying mechanisms by which evidence-informed decision-making might be achieved, the authors found reliable and effective evidence only for interventions facilitating access to research evidence and those building decision-makers’ skills to make sense of evidence. On the other hand, it also found reliable evidence of no effect for interventions that take a passive approach to communicating evidence, such as simple dissemination tools.

### Beyond communication and dissemination strategies

Many SRs in this area address questions not only about effectiveness but also about barriers, facilitators and mechanisms through which interventions affect outcomes [18, 19, 22, 27, 28, 31, 32].

There are a number of known barriers and enablers influencing the potential use of research evidence in decision-making at both the individual and organizational levels. Many other factors and mechanisms are still not sufficiently studied [52–55]. This complexity requires that strategies go beyond the simple “push” or “pull” of research evidence (communication and dissemination), combining them with strategies promoting processes of coproduction of knowledge, where decision-makers and researchers are interacting from the beginning, increasing the probability of translating research into policy [43, 45]. These multifaceted approaches include capacity-building workshops, policy dialogues or use of knowledge brokers, implemented through different structures such as KTPs [24, 56–59].

However, most of the evidence evaluating these multipronged strategies is based on case studies or similar methodologies, which makes it difficult to draw definitive conclusions about their effectiveness [24]. Likewise, the long-term sustainability of this type of intervention remains unclear. The few existing studies focus on outcomes more at the patient level than at the health system level or on policy impact [60]. Whatever facilitating process is adopted, it needs to recognize complexity and take into account that it often takes time, patience and multiple messages delivered through multiple channels before having an impact [54].

### Communication and dissemination in the time of COVID-19

Finally, a relevant and current issue is the use of evidence by decision-makers and managers in times of crisis, such as the current COVID-19 pandemic. In this context of public health emergency, there are three common challenges: (1) obstacles or barriers, for example, arising from uncertainty about risks of insufficient treatment or mistrust in the government, which will ultimately shape decisions and response measures; (2) variability in the way decision-makers acquire, interpret and apply evidence; and (3) limited capacity for decision-making because decision-makers often face competing demands on their time, limiting their ability to consider and act on available evidence [61].

We could also add that the current situation of COVID-19 is not only a “pandemic” but also an “infodemic”. Sufficient levels of knowledge are required not only to access, understand and act on relevant health information, but also to examine and assess the excessive and potentially misleading information in the wake of this infodemic. The forms of communication in this scenario are extremely relevant [62–64].

### Strengths and limitations of the overview

The main strength of our overview is that we followed a systematic and transparent process to locate and assess the evidence in this field using reliable methods and frameworks previously used by others, such as the taxonomy for classifying the communication and dissemination strategies [11], the AMSTAR 2 tool for assessing the methodological quality of reviews [12] and the data integration tables used for developing effectiveness statements [14].

However, our overview has a number of limitations. First, because of the project time frame, we have taken a “rapid overview” approach, implying that only part of the study selection and data extraction processes were performed in duplicate, which could have introduced bias and potential errors in our overview. Second, because we did not find an established intervention classification in the field (compared with what the Cochrane Effective Practice and Organisation of Care and Consumers and Communication Review groups have developed for intervention targeted at healthcare providers and consumers, respectively), we chose to adapt one previously developed by others [11]. Although the framework is aimed mainly at clinicians and patients, we felt it adequately fit for our overview goals. Third, because of the lack of standards regarding precise definitions of interventions and outcomes, we relied on our individual judgements when classifying the type of interventions and outcomes that each included review was focused on. In many cases, there were a number of interventions and outcomes mentioned in each of them, but we selected only those that were—in our opinion—the main focus of the review as established in the objectives and methods sections of each review. Fourth, because most of the evidence is presented in a narrative format, we were unable to evaluate the certainty of the evidence with established methodological approaches (such as GRADE [65]), and we used an alternative approach [14] (Additional file 4) that relied heavily on our judgements about the amount and strength of the evidence available in each review. To the best of our knowledge, while this approach has been used by others [3, 13], its reliability has not been validated. Lastly, the body of evidence in this field has a number of limitations not only at the evidence synthesis level but also with respect to the production of primary studies assessing the effectiveness of specific strategies. This production is limited by the challenges of implementing and assessing these types of interventions, which are embedded in complex health systems in which evidence is only one input to the decision-making process, and the effectiveness of communication and dissemination strategies could be confounded by a number of other factors [44]. In many cases it was not possible to disentangle the effects of the individual components of the interventions assessed, making the estimation of an effect size extremely difficult.

At the level of evidence synthesis, it is worth mentioning that most of the included reviews used combined strategies, but there was scarce information regarding details of characteristics such as the intensity, frequency, or duration of the interventions, as well as the way in which the information was presented. Researchers with ample experience in KT processes for policy recently designed a checklist to facilitate this. The checklist contains recommendations focused on items that quickly determine the relevance of the information and the key messages [66].

### Implications for research

Future research should examine the effectiveness of communication and dissemination strategies targeted at managers and policy-makers using sound methods and a more standardized and agreed upon set of outcomes, incorporating theoretical inputs into the intervention design.

This is especially urgent for interventions where we did not find sufficient direct evidence such as the use of different framings of the messages from research and the use of narratives to communicate research to decision-makers. Likewise, a better description of interventions in terms of type, intensity and duration will be needed in order to make reviews of primary studies more useful and relevant. More evidence is also needed on how the results or impacts of different interventions are measured to improve the uptake of knowledge. Many of the results evaluated were more subjective (self-reported) than objective. Further, the role of technology in this area should be explored in more detail. The use of technology-enhanced interventions is a promising area, especially in an era of remote work using digital interfaces.

Finally, an approximation or consensus on what would be the most appropriate framework for these strategies and contexts in which communication or dissemination activities are carried out would be desirable.

### Implications for policy

Because of the relative dearth of evidence about the effects of communication and dissemination strategies targeted at health managers and policy-makers for promoting the use of research evidence, it is not possible to make strong recommendations regarding the use of specific interventions. However, the targeting and tailoring of messages seems to be a key “ingredient” to be used in communication strategies, and the use of interventions that not only enhance recipients’ ability to use and apply evidence, but also motivate them to use and apply evidence (through leadership and social interactions), seems to be a key component of any dissemination strategy.

In addition, strategies to communicate and disseminate more broadly, not just push and pull mechanisms, should be considered. Although we have not found evidence of effectiveness for multifaceted strategies on changing behaviour, we think that combining them with those that promote, for example, knowledge coproduction processes, where decision-makers and researchers are interacting from the beginning, could increase the probability of translating research into policy. Some of these strategies have been promoted by KTPs such as those proposed by the Evidence-informed Policy Network (EVIPNet), and other initiatives such as the Structured Operational Research and Training IniTiative (SORT IT), which also include mentored capacity-building and knowledge coproduction and its implementation in the decision-making process.

On the other hand, the use of passive dissemination strategies aimed only at increasing the access and reach of the evidence does not seem to have a relevant effect on the knowledge or use of such evidence in decision-making processes. Additionally, when selecting any communication/dissemination strategy, a number of factors influencing the use and application of evidence should be carefully considered (what has been described as barriers and facilitators in this overview), identifying their presence in the specific settings where interventions will be implemented.

Considering the complexity of the decision-making environment in which managers and policy-makers perform their work, knowledge communication and dissemination strategies are just a single component of a multilevel evidence ecosystem/infrastructure that should be built in order to increase the role of evidence in decision-making.

## Conclusions

Knowledge communication strategies based on the use of targeted and tailored messages, and knowledge dissemination strategies that not only enhance recipients’ ability to use and apply evidence but also motivate them to do it, seem to be the more effective in promoting the use of evidence by managers and policy-makers. Passive dissemination strategies aimed only at increasing the access and reach of the evidence does not seem to have a relevant effect. However, there is a relative dearth of evidence about the effects of interventions targeting this audience compared to those targeting healthcare providers, consumers and patients. Also, more studies are needed that are informed by theoretical frameworks or specific tools, using robust methods, standardized outcome measures and clear descriptions of the interventions.

## Supplementary Information

**Additional file 1. **Search terms and results.

**Additional file 2. **Excluded studies with reasons.

**Additional file 3. **Characteristics of included studies.

**Additional file 4. **Evidence rating scheme.

**Additional file 5. **AMSTAR 2 quality assessment.

**Additional file 6. **Interventions and tools.

**Additional file 7. **Results and evidence statements.

**Additional file 8. **Frameworks/theories/models from included SRs.

## Data Availability

Data are available in the Additional file 1, Additional file 2, Additional file 3, Additional file 4, Additional file 5, Additional file 6, Additional file 7 and Additional file 8.

## Abbreviations

AMSTAR 2: A MeaSurement Tool to Assess systematic Reviews 2
EIPM: Evidence informed policy-making
EVIPNet: Evidence-informed Policy Network
KT: Knowledge translation
KTPs: Knowledge translation platforms
PRISMA: Preferred Reporting Items for Systematic Reviews and Meta-Analyses
SORT IT: Structured Operational Research and Training IniTiative
SRs: Systematic reviews
