# Supplementary material for: Assessing the impact of knowledge communication and dissemination strategies targeted at health policy-makers and managers: an overview of systematic reviews
Source: Health Res Policy Syst. 2021 Dec 6;19:140. doi: 10.1186/s12961-021-00780-4 (PMC8645346; doi:10.1186/s12961-021-00780-4)
Supplement: Supplementary file 1 — Additional file 1. Search terms and results. [file 12961_2021_780_MOESM1_ESM.docx]

## Additional file 1. Search terms and results

### Keyword areas for searching

| **Keyword area** | **Search terms** |
| --- | --- |
| Participants | ((polic* AND (maker* OR making* OR decision*)) OR "policy-makers" OR policymak* OR "policy-making" OR (decision* AND mak*) OR "decision-maker" OR "healthcare-administrator" OR "health-planner" OR "health-administrator" OR manager OR (program* AND (manage* OR officer OR director))) |
| Interventions | (diffusion* OR Competency-Based Education[Mesh] OR "competency-based education" OR "knowledge-dissemination" OR Knowledge Management[Mesh] OR "knowledge-management" OR "knowledge-diffusion" OR "knowledge-implementation" OR "knowledge-sharing" OR "knowledge-transfer" OR "knowledge-translation" OR "knowledge-broker" OR "evidence to policy" OR "research uptake" OR "research-uptake" OR "research use" OR "research-use" OR "use of research" OR "knowledge to action" OR "knowledge-to-action" OR "evidence-informed" OR "evidence informed" OR "research utilisation" OR "research utilization" OR "research communication" OR "research-communication" OR "research-dissemination" OR "research-diffusion" OR "research-implementation" OR "research-utilization" OR "research co-production" OR "knowledge-communication" OR "knowledge-exchange" OR "knowledge-mobilisation" OR "knowledge-mobilization" OR "knowledge uptake" OR "knowledge-uptake" OR "knowledge to policy") |
| Filter for Systematic review (Medline) | MEDLINE[tiab]  systematic review[Text Word]  meta-analysis[pt]  intervention*[ti] MEDLINE.tw  systematic review/  systematic review.tw.  meta-analysis/  intervention$.ti |

Keyword areas were combined using AND

### Search results from each source

| **Database** | **Date searched** | **No. refs found** | **No. refs included for full text** | **Final included (in progress)** |
| --- | --- | --- | --- | --- |
| **Electronic Databases** |  |  |  |  |
| CINAHL (EBSCOHost) | 01-02-21 | 205 | 7 | 1 |
| Embase | 01-02-21 | 713 | 9 | 3 |
| ERIC (EBSCOHost) | 03-02-21 | 22 | 2 | 1 |
| MEDLINE (PubMed) | 30-01-21 | 689 | 59 | 13 |
| PsycINFO (EBSCOHost) | 01-02-21 | 35 | 1 | 0 |
| World Wide Science | 02-02-21 | 259 | 2 | 1 |
| ***Specialized sources of systematic reviews*** |  |  |  |  |
| Cochrane Database of Systematic Reviews | 01-02-21 | 549 | 2 | 1 |
| Epistemonikos (already include the following databases): | 01-02-21 | 154 | 5 | 4 |
| -      The Campbell Collaboration |  |  |  |  |
| -      EPPI-Centre Database of Health Promotion Research (BiblioMap) |  |  |  |  |
| -      Database of Promoting Health Effectiveness Reviews (DoPHER) |  |  |  |  |
| -      LILACS |  |  |  |  |
| -      Database of Abstracts of Reviews of Effects |  |  |  |  |
| Health Systems Evidence | 03-02-21 | 348 | 0 | 0 |
| PDQ-Evidence | 03-02-21 | 14 | 0 | 0 |
| Total: |  | 2988 | 87 | 24 |
| **Manual searches** |  |  |  |  |
| Google and Google Scholar (11 first pages) | 03-02-21 | 16 | 16 | 2 |
| [Rx](https://www.cadth.ca/rx-change) for Change | 03-02-21 | 21 | 0 | 0 |
| 3ie - International Initiative for Impact Evaluation | 03-02-21 | 33 | 0 | 0 |
| Institut of education sciences | 03-02-21 | 73 | 0 | 0 |
| References list | 18-02-21 |  | 2 | 1 |
| Total: |  | 127 | 18 | 3 |
| **TOTAL:** |  | **3115** | **105** | **27** |

Search for primary studies in MEDLINE (PubMed) on

## Figure 1 - Study selection flow chart

### Search strategies

**CINAHL – 01 Feb 2021**

| **#** | **Query** | **Results** |
| --- | --- | --- |
| S1 | ((polic* AND (maker* OR making* OR decision*)) | 47.949 |
| S2 | "policy-makers" | 12.247 |
| S3 | policymak* | 7.170 |
| S4 | "policy-making" | 17.175 |
| S5 | (decision* AND mak*) | 165.845 |
| S6 | "decision-maker" | 641 |
| S7 | "healthcare-administrator" | 4 |
| S8 | "health-planner" | 8 |
| S9 | "health-administrator" | 27 |
| S10 | manager | 44.380 |
| S11 | (program* AND (manage* OR officer OR director))) | 94.435 |
| S12 | S1 OR S2 OR S3 OR S4 OR S5 OR S6 OR S7 OR S8 OR S9 OR S10 OR S11 | **321.570** |
| S13 | diffusion* | 34.503 |
| S14 | Competency-Based Education MH | 14.408 |
| S15 | "competency-based education" | 1.818 |
| S16 | "knowledge-dissemination" | 194 |
| S17 | Knowledge Management MH | 2.232 |
| S18 | "knowledge-management" | 2.664 |
| S19 | "knowledge-diffusion" | 30 |
| S20 | "knowledge-implementation" | 41 |
| S21 | "knowledge-sharing" | 683 |
| S22 | "knowledge-transfer" | 898 |
| S23 | "knowledge-translation" | 1.938 |
| S24 | "knowledge-broker" | 53 |
| S25 | "evidence to policy" | 985 |
| S26 | "research uptake" | 42 |
| S27 | "research-uptake" | 42 |
| S28 | "research use" | 574 |
| S29 | "research-use" | 574 |
| S30 | "use of research" | 2.060 |
| S31 | "knowledge to action" | 440 |
| S32 | "knowledge-to-action" | 440 |
| S33 | "evidence-informed" | 1.630 |
| S34 | "evidence informed" | 1.630 |
| S35 | "research utilisation" | 113 |
| S36 | "research utilization" | 1.625 |
| S37 | "research communication" | 61 |
| S38 | "research-communication" | 61 |
| S39 | "research-dissemination" | 181 |
| S40 | "research-diffusion" | 6 |
| S41 | "research-implementation" | 298 |
| S42 | "research-utilization" | 1.625 |
| S43 | "research co-production" | 8 |
| S44 | "knowledge-communication" | 126 |
| S45 | "knowledge-exchange" | 410 |
| S46 | "knowledge-mobilisation" | 25 |
| S47 | "knowledge-mobilization" | 74 |
| S48 | "knowledge uptake" | 43 |
| S49 | "knowledge-uptake" | 43 |
| S50 | "knowledge to policy" | 82 |
| S51 | S13 OR S14 OR S15 OR S16 OR S17 OR S18 OR S19 OR S20 OR S21 OR S22 OR S23 OR S24 OR S25 OR S26 OR S27 OR S28 OR S29 OR S30 OR S31 OR S32 OR S33 OR S34 OR S35 OR S36 OR S37 OR S38 OR S39 OR S40 OR S41 OR S42 OR S43 OR S44 OR S45 OR S46 OR S47 OR S48 OR S49 OR S50 | **48.971** |
| S52 | (PT systematic review OR meta-analysis) | **108.335** |
| S53 | S12 AND S51 AND S52 (Limited 2006-2021) | **205** |

**EMBASE (Ovid) – 01 Feb. 2021**

| #1 | ((polic* AND (maker* OR making* OR decision*)) | 118,683 |
| --- | --- | --- |
| #2 | policy-makers' | 28,288 |
| #3 | policymak* | 15,830 |
| #4 | policy-making' | 6,143 |
| #5 | (decision* AND mak*) | 527,279 |
| #6 | decision-maker' | 17 |
| #7 | healthcare-administrator' | 13 |
| #8 | health-planner' | 50 |
| #9 | health-administrator' | 97 |
| #10 | manager | 51.982 |
| #11 | (program* AND (manage* OR officer OR director))) | 382.581 |
| #12 | #1 OR #2 OR #3 OR #4 OR #5 OR #6 OR #7 OR #8 OR #9 OR #10 OR #11 | 987.154 |
| #13 | diffusion* | 314.082 |
| #14 | curriculum'/exp | 94.486 |
| #15 | competency-based education' | 901 |
| #16 | knowledge-dissemination' | 474 |
| #17 | Knowledge Management'/exp | 1.761 |
| #18 | knowledge-management' | 3.490 |
| #19 | knowledge-diffusion' | 77 |
| #20 | knowledge-implementation' | 95 |
| #21 | knowledge-sharing' | 1.138 |
| #22 | knowledge-transfer' | 3.078 |
| #23 | knowledge-translation' | 5.556 |
| #24 | knowledge-broker' | 114 |
| #25 | evidence to policy' | 141 |
| #26 | research uptake' | 97 |
| #27 | research-uptake' | 97 |
| #28 | research use' | 2.284 |
| #29 | research-use' | 2.284 |
| #30 | use of research' | 1.021 |
| #31 | knowledge to action' | 441 |
| #32 | knowledge-to-action' | 441 |
| #33 | evidence-informed' | 3.123 |
| #34 | evidence informed' | 3.123 |
| #35 | research utilisation' | 115 |
| #36 | research utilization' | 854 |
| #37 | research communication' | 1.305 |
| #38 | research-communication' | 1305 |
| #39 | research-dissemination' | 443 |
| #40 | research-diffusion' | 18 |
| #41 | research-implementation' | 556 |
| #42 | research-utilization' | 854 |
| #43 | research co-production' | 16 |
| #44 | knowledge-communication' | 252 |
| #45 | knowledge-exchange' | 968 |
| #46 | knowledge-mobilisation' | 69 |
| #47 | knowledge-mobilization' | 170 |
| #48 | knowledge uptake' | 120 |
| #49 | knowledge-uptake' | 120 |
| #50 | knowledge to policy' | 41 |
| #51 | #13 OR #14 OR #15 OR #16 OR #17 OR #18 OR #19 OR #20 OR #21 OR #22 OR #23 OR #24 OR #25 OR #26 OR #27 OR #28 OR #29 OR #30 OR #31 OR #32 OR #33 OR #34 OR #35 OR #36 OR #37 OR #38 OR #39 OR #40 OR #41 OR #42 OR #43 OR #44 OR #45 OR #46 OR #47 OR #48 OR #49 OR #50 | 431.668 |
| #52 | #12 AND #51 | 29.062 |
| #53 | #12 AND #51 AND ([systematic review]/lim OR [metaanalysis]/lim) AND [2006-2021]/PY | **713** |

**ERIC – 03 Feb. 2021**

| **#** | **Query** | **Results** |
| --- | --- | --- |
| **1** | (kw: polic* AND (kw: maker* OR kw: making* OR kw: decision*)) | 36716 |
| **2** | kw: policy-makers | 1816 |
| **3** | kw: policymak* | 30474 |
| **4** | kw: policy-making | 1535 |
| **5** | kw: decision* AND kw: mak* | 71683 |
| **6** | kw: decision-maker | 0 |
| **7** | kw: healthcare-administrator | 0 |
| **8** | kw: health-planner | 0 |
| **9** | kw: health-administrator | 0 |
| **10** | kw: manager | 3852 |
| **11** | (kw: program* AND (kw: manage* OR kw: officer OR kw: director)) | 66426 |
| **12** | 1 OR 2 OR 3 OR 4 OR 5 OR 6 OR 7 OR 8 OR 9 OR 10 OR 11 | 177842 |
| **13** | kw: diffusion* | 4289 |
| **14** | kw: competency-based and kw: education | 4755 |
| **15** | kw: knowledge-dissemination | 0 |
| **16** | su= "Knowledge Management" | 1819 |
| **17** | kw: knowledge-management | 19 |
| **18** | kw: knowledge-diffusion | 1 |
| **19** | kw: "knowledge-implementation" | 1 |
| **20** | kw: knowledge-implementation | 1 |
| **21** | kw: knowledge-transfer | 16 |
| **22** | kw: knowledge-translation | 6 |
| **23** | kw: knowledge-broker | 1 |
| **24** | kw: "evidence to policy" | 437 |
| **25** | kw: research and kw: uptake | 10 |
| **26** | kw: "research-uptake" | 0 |
| **27** | kw: research and kw: use | 204 |
| **28** | kw: "research-use" | 1 |
| **29** | kw: "use of research" | 1422 |
| **30** | kw: "knowledge to action" | 288 |
| **31** | kw: "knowledge-to-action" | 10 |
| **32** | kw: "evidence-informed" | 253 |
| **33** | kw: evidence and kw: informed | 270 |
| **34** | kw: "research and kw:utilisation" | 17 |
| **35** | kw: research and kw: utilization | 5629 |
| **36** | kw: research and kw: communication | 61725 |
| **37** | kw: "research-communication" | 0 |
| **38** | kw: "research-dissemination" | 2 |
| **39** | kw: "research-diffusion" | 0 |
| **40** | kw: "research-implementation" | 1 |
| **41** | kw: "research-utilization" | 1 |
| **42** | kw: research and kw: co-production | 74 |
| **43** | kw: "knowledge-communication" | 0 |
| **44** | kw: "knowledge-exchange" | 5 |
| **45** | kw: "knowledge-mobilisation" | 0 |
| **46** | kw: "knowledge-mobilization" | 0 |
| **47** | kw: knowledge and kw: uptake. | 236 |
| **48** | kw: "knowledge-uptake" | 0 |
| **49** | kw: "knowledge to policy" | 14160 |
| **50** | 13 OR 14 OR 15 OR 16 OR 17 OR 18 OR 19 OR 20 OR 21 OR 22 OR 23 OR 24 OR 25 OR 26 OR 27 OR 28 OR 29 OR 30 OR 31 OR 32 OR 33 OR 34 OR 35 OR 36 OR 37 OR 38 OR 39 OR 40 OR 41 OR 42 OR 43 OR 44 OR 45 OR 46 OR 47 OR 48 OR 49 | 12983 |
| **51** | 12 AND 50 | 1565 |
| **52** | #12 AND #50 ["systematic review" or meta-analysis or metanalysis and yr: 2006-2021] | **22** |

**MEDLINE (PubMed) – 30 Jan. 2021**

| #1 | (polic* AND (maker* OR making* OR decision*)) | 102.330 |
| --- | --- | --- |
| #2 | "policy-makers" | 24.116 |
| #3 | policymak* | 22.014 |
| #4 | "policy-making" | 20.772 |
| #5 | (decision* AND mak*) | 1.071 |
| #6 | "decision-maker" | 7 |
| #7 | "healthcare-administrator" | 13 |
| #8 | "health-planner" | 33 |
| #9 | "health-administrator" | 91 |
| #10 | manager | 3.171.121 |
| #11 | (program* AND (manage* OR officer OR director)) | 262.952 |
| #12 | #1 OR #2 OR #3 OR #4 OR #5 OR #6 OR #7 OR #8 OR #9 OR #10 OR #11 | 3.267.860 |
| #13 | diffusion* | 247.697 |
| #14 | Competency-Based Education[Mesh] | 4.096 |
| #15 | "competency-based education" | 4.513 |
| #16 | "knowledge-dissemination" | 337 |
| #17 | Knowledge Management[Mesh] | 369 |
| #18 | "knowledge-management" | 2.015 |
| #19 | "knowledge-diffusion" | 60 |
| #20 | "knowledge-implementation" | 74 |
| #21 | "knowledge-sharing" | 888 |
| #22 | "knowledge-transfer" | 2.379 |
| #23 | "knowledge-translation" | 4.302 |
| #24 | "knowledge-broker" | 92 |
| #25 | "evidence to policy" | 131 |
| #26 | "research uptake" | 81 |
| #27 | "research-uptake" | 81 |
| #28 | "research use" | 1.408 |
| #29 | "research-use" | 1.408 |
| #30 | "use of research" | 991 |
| #31 | "knowledge to action" | 340 |
| #32 | "knowledge-to-action" | 340 |
| #33 | "evidence-informed" | 2.557 |
| #34 | "evidence informed" | 2.557 |
| #35 | "research utilisation" | 105 |
| #36 | "research utilization" | 761 |
| #37 | "research communication" | 308 |
| #38 | "research-communication" | 308 |
| #39 | "research-dissemination" | 331 |
| #40 | "research-diffusion" | 0 |
| #41 | "research-implementation" | 485 |
| #42 | "research-utilization" | 761 |
| #43 | "research co-production" | 15 |
| #44 | "knowledge-communication" | 179 |
| #45 | "knowledge-exchange" | 746 |
| #46 | "knowledge-mobilisation" | 75 |
| #47 | "knowledge-mobilization" | 122 |
| #48 | "knowledge uptake" | 72 |
| #49 | "knowledge-uptake" | 72 |
| #50 | "knowledge to policy" | 19 |
| #51 | #13 OR #14 OR #15 OR #16 OR #17 OR #18 OR #19 OR #20 OR #21 OR #23 OR #24 OR #25 OR #26 OR #27 OR #28 OR #29 OR #30 OR #31 OR #32 OR #33 OR #34 OR #35 OR #36 OR #37 OR #38 OR #39 OR #40 OR #41 OR #42 OR #43 OR #44 OR #45 OR #46 OR #47 OR #48 OR #49 OR #50 | 268.138 |
| #52 | #12 AND #52 | 31.448 |
| #53 | Filters: Meta-Analysis, Systematic Review, from 2006 - 2021 | **689** |

**PsycINFO (EBSCOHost) – 01 Feb. 2021**

| S1 | ((polic* AND (maker* OR making* OR decision*)) | 86.562 |
| --- | --- | --- |
| S2 | "policy-makers" | 16.070 |
| S3 | policymak* | 19.723 |
| S4 | "policy-making" | 46.205 |
| S5 | (decision* AND mak*) | 166.187 |
| S6 | "decision-maker" | 546 |
| S7 | "healthcare-administrator" | 7 |
| S8 | "health-planner" | 10 |
| S9 | "health-administrator" | 34 |
| S10 | manager | 58.580 |
| S11 | (program* AND (manage* OR officer OR director))) | 83.993 |
| S12 | S1 OR S2 OR S3 OR S4 OR S5 OR S6 OR S7 OR S8 OR S9 OR S10 OR S11 | 27.628 |
| S13 | diffusion* | 22.227 |
| S14 | MA Competency-Based Education | 448 |
| S15 | "competency-based education" | 660 |
| S16 | "knowledge-dissemination" | 242 |
| S17 | MA Knowledge Management | 48 |
| S18 | "knowledge-management" | 5.808 |
| S19 | "knowledge-diffusion" | 91 |
| S20 | "knowledge-implementation" | 40 |
| S21 | "knowledge-sharing" | 2.495 |
| S22 | "knowledge-transfer" | 3.892 |
| S23 | "knowledge-translation" | 983 |
| S24 | "knowledge-broker" | 43 |
| S25 | "evidence to policy" | 974 |
| S26 | "research uptake" | 27 |
| S27 | "research-uptake" | 27 |
| S28 | "research use" | 522 |
| S29 | "research-use" | 522 |
| S30 | "use of research" | 3.010 |
| S31 | "knowledge to action" | 569 |
| S32 | "knowledge-to-action" | 569 |
| S33 | "evidence-informed" | 1.345 |
| S34 | "evidence informed" | 1.345 |
| S35 | "research utilisation" | 59 |
| S36 | "research utilization" | 379 |
| S37 | "research communication" | 104 |
| S38 | "research-communication" | 104 |
| S39 | "research-dissemination" | 208 |
| S40 | "research-diffusion" | 6 |
| S41 | "research-implementation" | 198 |
| S42 | "research-utilization" | 379 |
| S43 | "research co-production" | 1 |
| S44 | "knowledge-communication" | 147 |
| S45 | "knowledge-exchange" | 553 |
| S46 | "knowledge-mobilisation" | 27 |
| S47 | "knowledge-mobilization" | 95 |
| S48 | "knowledge uptake" | 25 |
| S49 | "knowledge-uptake" | 25 |
| S50 | "knowledge to policy" | 140 |
| S51 | S13 OR S14 OR S15 OR S16 OR S17 OR S18 OR S19 OR S20 OR S21 OR S22 OR S23 OR S24 OR S25 OR S26 OR S27 OR S28 OR S29 OR S30 OR S31 OR S32 OR S33 OR S34 OR S35 OR S36 OR S37 OR S38 OR S39 OR S40 OR S41 OR S42 OR S43 OR S44 OR S45 OR S46 OR S47 OR S48 OR S49 OR S50 | 40.701 |
| S52 | (PT systematic review OR meta-analysis) | 38.052 |
| S53 | S12 AND S51 AND S52 (Lim. year 2006-2021) | **35** |

**World Wide Science – 02 Feb. 2021**

| 1 | (polic* AND (maker* OR making* OR decision*)) | 989 |
| --- | --- | --- |
| 2 | "policy-makers" | 1862 |
| 3 | policymak* | 1379 |
| 4 | "policy-making" | 2206 |
| 5 | (decision* AND mak*) | 960 |
| 6 | "decision-maker" | 386 |
| 7 | "healthcare-administrator" | 672 |
| 8 | "health-planner" | 631 |
| 9 | "health-administrator" | 873 |
| 10 | manager | 2337 |
| 11 | (program* AND (manage* OR officer OR director)) | 1144 |
| 12 | #1 OR #2 OR #3 OR #4 OR #5 OR #6 OR #7 OR #8 OR #9 OR #10 OR #11 | 1047 |
| 13 | diffusion* | 1773 |
| 14 | "competency-based education" | 1329 |
| 15 | "knowledge-dissemination" | 1573 |
| 16 | "knowledge management" | 1667 |
| 17 | "knowledge-management" | 1618 |
| 18 | "knowledge-diffusion" | 1465 |
| 19 | "knowledge-implementation" | 1230 |
| 20 | "knowledge-sharing" | 1544 |
| 21 | "knowledge-transfer" | 1995 |
| 22 | "knowledge-translation" | 667 |
| 23 | "knowledge-broker" | 763 |
| 24 | "evidence to policy" | 624 |
| 25 | "research uptake" | 1144 |
| 26 | "research-uptake" | 996 |
| 27 | "research use" | 2565 |
| 28 | "research-use" | 2236 |
| 29 | "use of research" | 2127 |
| 30 | "knowledge to action" | 1476 |
| 31 | "knowledge-to-action" | 1106 |
| 32 | "evidence-informed" | 1327 |
| 33 | "evidence informed" | 1154 |
| 34 | "research utilisation" | 1167 |
| 35 | "research utilization" | 1144 |
| 36 | "research communication" | 1714 |
| 37 | "research-communication" | 1921 |
| 38 | "research-dissemination" | 1355 |
| 39 | "research-diffusion" | 878 |
| 40 | "research-implementation" | 1184 |
| 41 | "research-utilization" | 1800 |
| 42 | "research co-production" | 935 |
| 43 | "knowledge-communication" | 1733 |
| 44 | "knowledge-exchange" | 1727 |
| 45 | "knowledge-mobilisation" | 974 |
| 46 | "knowledge-mobilization" | 1092 |
| 47 | "knowledge uptake" | 658 |
| 48 | "knowledge-uptake" | 1123 |
| 49 | "knowledge to policy" | 767 |
| 50 | 13 OR 14 OR 15 OR 16 OR 17 OR 18 OR 19 OR 20 OR 21 OR 22 OR 23 OR 24 OR 25 OR 26 OR 27 OR 28 OR 29 OR 30 OR 31 OR 31 OR 32 OR 33 OR 34 OR 35 OR 36 OR 37 OR 38 OR 39 OR 40 OR 41 OR 42 OR 43 OR 44 OR 45 OR 46 OR 47 OR 48 OR 49 OR 50 | 899 |
| 51 | #12 AND #50 /FROM: 2006 TO: 2021 | **259** |

***Specialized sources of systematic reviews***

**Cochrane Library (Cochrane Database of Systematic Reviews, DARE, HTA) – 01 Feb. 2021**

| #1 | ((polic* AND (maker* OR making* OR decision*)) | 5.329 |
| --- | --- | --- |
| #2 | "policy-makers" | 1.853 |
| #3 | policymak* | 1.118 |
| #4 | "policy-making" | 243 |
| #5 | (decision* AND mak*) | 22.761 |
| #6 | "decision-maker" | 1 |
| #7 | "healthcare-administrator" | 1 |
| #8 | "health-planner" | 2 |
| #9 | "health-administrator" | 2 |
| #10 | manager | 8.306 |
| #11 | (program* AND (manage* OR officer OR director))) | 30.437 |
| #12 | {OR #1-#11} | 52.779 |
| #13 | diffusion* | 4.403 |
| #14 | MeSH descriptor: [Competency-Based Education] explode all trees | 85 |
| #15 | "competency-based education" | 1 |
| #16 | "knowledge-dissemination" | 114 |
| #17 | MeSH descriptor: [Knowledge Management] explode all trees | 7 |
| #18 | "knowledge-management" | 55 |
| #19 | "knowledge-diffusion" | 1 |
| #20 | "knowledge-implementation" | 8 |
| #21 | "knowledge-sharing" | 29 |
| #22 | "knowledge-transfer" | 235 |
| #23 | "knowledge-translation" | 486 |
| #24 | "knowledge-broker" | 9 |
| #25 | "evidence to policy" | 13 |
| #26 | "research uptake" | 6 |
| #27 | "research-uptake" | 6 |
| #28 | "research use" | 144 |
| #29 | "research-use" | 144 |
| #30 | "use of research" | 67 |
| #31 | "knowledge to action" | 32 |
| #32 | "knowledge-to-action" | 32 |
| #33 | "evidence-informed" | 324 |
| #34 | "evidence informed" | 327 |
| #35 | "research utilisation" | 30 |
| #36 | "research utilization" | 30 |
| #37 | "research communication" | 14 |
| #38 | "research-communication" | 14 |
| #39 | "research-dissemination" | 25 |
| #40 | "research-diffusion" | 2 |
| #41 | "research-implementation" | 56 |
| #42 | "research-utilization" | 30 |
| #43 | "research co-production" | 1 |
| #44 | "knowledge-communication" | 25 |
| #45 | "knowledge-exchange" | 31 |
| #46 | "knowledge-mobilisation" | 3 |
| #47 | "knowledge-mobilization" | 3 |
| #48 | "knowledge uptake" | 15 |
| #49 | "knowledge-uptake" | 15 |
| #50 | "knowledge to policy" | 0 |
| #51 | {OR #13- #50} | 5.971 |
| #52 | #12 AND #51 | 1.069 |
| #53 | #12 AND #52 [with Cochrane Library publication date from Jan 2006 to Dec 2021] | 1.018 |
|  | [Only SRs and protocols] | **549** |

**Epistemonikos – 01 Feb. 2021**

| 1 | (polic* AND (maker* OR making* OR decision*)) | 5,450 |
| --- | --- | --- |
| 2 | "policy-makers" | 642 |
| 3 | policymak* | 1736 |
| 4 | "policy-making" | 207 |
| 5 | (decision* AND mak*) | 10186 |
| 6 | "decision-maker" | 60 |
| 7 | "healthcare-administrator" | 0 |
| 8 | "health-planner" | 0 |
| 9 | "health-administrator" | 0 |
| 10 | manager | 4265 |
| 11 | (program* AND (manage* OR officer OR director)) | 10229 |
| 12 | 1 OR 2 OR 3 OR 4 OR 5 OR 6 OR 7 OR 8 OR 9 OR 10 OR 11 | 127774 |
| 13 | diffusion* | 2902 |
| 14 | "competency-based education" | 27 |
| 15 | "knowledge-dissemination" | 0 |
| 16 | "knowledge management" | 82 |
| 17 | "knowledge-management" | 1 |
| 18 | "knowledge-diffusion" | 0 |
| 19 | "knowledge-implementation" | 0 |
| 20 | "knowledge-sharing" | 15 |
| 21 | "knowledge-transfer" | 3 |
| 22 | "knowledge-translation" | 2 |
| 23 | "knowledge-broker" | 0 |
| 24 | "evidence to policy" | 14 |
| 25 | "research uptake" | 9 |
| 26 | "research-uptake" | 0 |
| 27 | "research use" | 95 |
| 28 | "research-use" | 2 |
| 29 | "use of research" | 132 |
| 30 | "knowledge to action" | 9 |
| 31 | "knowledge-to-action" | 9 |
| 32 | "evidence-informed" | 258 |
| 33 | "evidence informed" | 44 |
| 34 | "research utilisation" | 12 |
| 35 | "research utilization" | 78 |
| 36 | "research communication" | 8 |
| 37 | "research-communication" | 0 |
| 38 | "research-dissemination" | 0 |
| 39 | "research-diffusion" | 0 |
| 40 | "research-implementation" | 1 |
| 41 | "research-utilization" | 0 |
| 42 | "research co-production" | 0 |
| 43 | "knowledge-communication" | 0 |
| 44 | "knowledge-exchange" | 0 |
| 45 | "knowledge-mobilisation" | 0 |
| 46 | "knowledge-mobilization" | 0 |
| 47 | "knowledge uptake" | 10 |
| 48 | "knowledge-uptake" | 1 |
| 49 | "knowledge to policy" | 1 |
| 50 | 13 OR 14 OR 15 OR 16 OR 17 OR 18 OR 19 OR 20 OR 21 OR 22 OR 23 OR 24 OR 25 OR 26 OR 27 OR 28 OR 29 OR 30 OR 31 OR 32 OR 33 OR 34 OR 35 OR 36 OR 37 OR 38 OR 39 OR 40 OR 41 OR 42 OR 43 OR 44 OR 45 OR 46 OR 47 OR 48 OR 49 | 3616 |
| 51 | 12 AND 50 | 332 |
| 52 | 12 AND 50 [date range: 2006-2021 and type document: systematic review] | **154** |

**Health Systems Evidence – 03 Feb. 2021**

| 1 | ((polic* AND (maker* OR making* OR decision*)) | 1785 |
| --- | --- | --- |
| 2 | "policy-makers" | 327 |
| 3 | policymak* | 156 |
| 4 | "policy-making" | 59 |
| 5 | (decision* AND mak*) | 2654 |
| 6 | "decision-maker" | 22 |
| 7 | "healthcare-administrator" | 0 |
| 8 | "health-planner" | 0 |
| 9 | "health-administrator" | 0 |
| 10 | manager | 1263 |
| 11 | (program* AND (manage* OR officer OR director)) | 7411 |
| 12 | 1 OR 2 OR 3 OR 4 OR 5 OR 6 OR 7 OR 8 OR 9 OR 10 OR 11 | 9020 |
| 13 | diffusion* | 861 |
| 14 | "competency-based education" | 11 |
| 15 | "knowledge-dissemination" | 2 |
| 16 | "knowledge management" | 797 |
| 17 | "knowledge-management" | 797 |
| 18 | "knowledge-diffusion" | 0 |
| 19 | "knowledge-implementation" | 0 |
| 20 | "knowledge-sharing" | 8 |
| 21 | "knowledge-transfer" | 20 |
| 22 | "knowledge-translation" | 59 |
| 23 | "knowledge-broker" | 3 |
| 24 | "evidence to policy" | 1 |
| 25 | "research uptake" | 1 |
| 26 | "research-uptake" | 1 |
| 27 | "research use" | 11 |
| 28 | "research-use" | 11 |
| 29 | "use of research" | 19 |
| 30 | "knowledge to action" | 1 |
| 31 | "knowledge-to-action" | 1 |
| 32 | "evidence-informed" | 585 |
| 33 | "evidence informed" | 585 |
| 34 | "research utilisation" | 0 |
| 35 | "research utilization" | 4 |
| 36 | "research communication" | 1 |
| 37 | "research-communication" | 1 |
| 38 | "research-dissemination" | 2 |
| 39 | "research-diffusion" | 0 |
| 40 | "research-implementation" | 5 |
| 41 | "research-utilization" | 4 |
| 42 | "research co-production" | 0 |
| 43 | "knowledge-communication" | 3 |
| 44 | "knowledge-exchange" | 11 |
| 45 | "knowledge-mobilisation" | 2 |
| 46 | "knowledge-mobilization" | 0 |
| 47 | "knowledge uptake" | 3 |
| 48 | "knowledge-uptake" | 3 |
| 49 | "knowledge to policy" | 4 |
| 50 | 13 OR 14 OR 15 OR 16 OR 17 OR 18 OR 19 OR 20 OR 21 OR 22 OR 23 OR 24 OR 25 OR 26 OR 27 OR 28 OR 29 OR 30 OR 31 OR 32 OR 33 OR 34 OR 35 OR 36 OR 37 OR 38 OR 39 OR 40 OR 41 OR 42 OR 43 OR 44 OR 45 OR 46 OR 47 OR 48 OR 49 | 1670 |
| 51 | #12 AND #50 [systematic reviews on effects - date range: 2006-2021] | **348** |

**PDQ-Evidence – 03 Feb. 2021**

| 1 | (polic* AND (maker* OR making* OR decision*)) | 841 |
| --- | --- | --- |
| 2 | "policy-makers" | 91 |
| 3 | policymak* | 210 |
| 4 | "policy-making" | 24 |
| 5 | (decision* AND mak*) | 1603 |
| 6 | "decision-maker" | 10 |
| 7 | "healthcare-administrator" | 0 |
| 8 | "health-planner" | 0 |
| 9 | "health-administrator" | 0 |
| 10 | manager | 326 |
| 11 | (program* AND (manage* OR officer OR director)) | 3046 |
| 12 | 1 OR 2 OR 3 OR 4 OR 5 OR 6 OR 7 OR 8 OR 9 OR 10 OR 11 | 5535 |
| 13 | diffusion* | 141 |
| 14 | "competency-based education" | 0 |
| 15 | "knowledge-dissemination" | 0 |
| 16 | "knowledge management" | 21 |
| 17 | "knowledge-management" | 0 |
| 18 | "knowledge-diffusion" | 0 |
| 19 | "knowledge-implementation" | 0 |
| 20 | "knowledge-sharing" | 1 |
| 21 | "knowledge-transfer" | 2 |
| 22 | "knowledge-translation" | 1 |
| 23 | "knowledge-broker" | 0 |
| 24 | "evidence to policy" | 3 |
| 25 | "research uptake" | 3 |
| 26 | "research-uptake" | 0 |
| 27 | "research use" | 17 |
| 28 | "research-use" | 0 |
| 29 | "use of research" | 22 |
| 30 | "knowledge to action" | 1 |
| 31 | "knowledge-to-action" | 1 |
| 32 | "evidence-informed" | 23 |
| 33 | "evidence informed" | 4 |
| 34 | "research utilisation" | 3 |
| 35 | "research utilization" | 22 |
| 36 | "research communication" | 0 |
| 37 | "research-communication" | 0 |
| 38 | "research-dissemination" | 0 |
| 39 | "research-diffusion" | 0 |
| 40 | "research-implementation" | 0 |
| 41 | "research-utilization" | 0 |
| 42 | "research co-production" | 0 |
| 43 | "knowledge-communication" | 0 |
| 44 | "knowledge-exchange" | 0 |
| 45 | "knowledge-mobilisation" | 0 |
| 46 | "knowledge-mobilization" | 0 |
| 47 | "knowledge uptake" | 5 |
| 48 | "knowledge-uptake" | 0 |
| 49 | "knowledge to policy" | 0 |
| 50 | 13 OR 14 OR 15 OR 16 OR 17 OR 18 OR 19 OR 20 OR 21 OR 22 OR 23 OR 24 OR 25 OR 26 OR 27 OR 28 OR 29 OR 30 OR 31 OR 32 OR 33 OR 34 OR 35 OR 36 OR 37 OR 38 OR 39 OR 40 OR 41 OR 42 OR 43 OR 44 OR 45 OR 46 OR 47 OR 48 OR 49 | 249 |
| 51 | 12 AND 50 | 66 |
| 52 | 12 AND 50 [date range: 2006-2021 and type document: systematic review] | **14** |

**Manual searches**

RX for change 03 Feb. 2021

Search Strategy:

| #1 | policy-maker* AND "Knowledge Management" | 19 |
| --- | --- | --- |
| #2 | date range: 2006-2021 | 21 |

International Initiative on Impact Evaluation- 03 Feb. 2021

Database: International Initiative on Impact Evaluation <2006 to **03 Feb. 2021**>

Search Strategy:

| #1 | police | 7 |
| --- | --- | --- |
| #2 | abstract:(Knowledge Diffusion) | 342 |
| #3 | abstract:(knowledge traslatation) | 355 |
| #4 | abstract:(knowledge sharing) | 360 |
| #5 | abstract:(knowledge transfer) | 580 |
| #6 | #2 OR #3 OR #4 OR #5 | 610 |
| #7 | #1 AND #6 | 35 |
|  | Lim. date range 2006-2021 | **33** |

IES Institute of education sciences - 04 Feb. 2021

| #1 | (police-makers OR police-making) | 1.092 |
| --- | --- | --- |
| #2 | (knowledge-management OR knowledge-sharing OR knowledge-diffusion) | 1.152 |
| #3 | #1 AND #2 | 2.611 |
| #4 | Lim 2006-2021+ quick review [Type of product] | **73** |
