# Supplementary material for: Assessing the impact of knowledge communication and dissemination strategies targeted at health policy-makers and managers: an overview of systematic reviews
Source: Health Res Policy Syst. 2021 Dec 6;19:140. doi: 10.1186/s12961-021-00780-4 (PMC8645346; doi:10.1186/s12961-021-00780-4)
Supplement: Supplementary file 2 — Additional file 2. Excluded studies with reasons. [file 12961_2021_780_MOESM2_ESM.docx]

Additional file 2. Excluded studies with reasons.

| Reference | Reason |
| --- | --- |
| 1. Abdullah G, Rossy D, Ploeg J, Davies B, Higuchi K, sikora L, et al. Measuring the effectiveness of mentoring as a knowledge translation intervention for implementing empirical evidence: a systematic review. Worldviews Evid Based Nurs. 2014;11(5):284-300. | Not targeted at policymakers or managers |
| 1. Akl EA, Fadlallah R, Ghandour L, Kdouh O, Langlois E, Lavis JN, et al. The SPARK Tool to prioritise questions for systematic reviews in health policy and systems research: Development and initial validation. Health Research Policy and Systems. 2017;15(1). | Not a systematic review of effects/impact |
| 1. Akl EA, Oxman AD, Herrin J, Vist GE, Terrenato I, Sperati F, Costiniuk C, Blank D, Schünemann H. Using alternative statistical formats for presenting risks and risk reductions. Cochrane Database of Systematic Reviews 2011, Issue 3. Art. No.: CD006776. DOI: 10.1002/14651858.CD006776.pub2. | Not targeted at policymakers or managers |
| 1. Alla K, Oprescu F, Hall WD, Whiteford HA, Head BW, Meurk CS. Can automated content analysis be used to assess and improve the use of evidence in mental health policy? A systematic review. Syst Rev. 2018;7(1):194. | Not a knowledge communication/dissemination strategy |
| 1. Allen JD, Towne SD Jr, Maxwell AE, DiMartino L, Leyva B, Bowen DJ, Linnan L, Weiner BJ. Meausures of organizational characteristics associated with adoption and/or implementation of innovations: A systematic review. BMC Health Serv Res. 2017 Aug 23;17(1):591. doi: 10.1186/s12913-017-2459-x. PMID: 28835273; PMCID: PMC5569532. | Not targeted at policymakers or managers |
| 1. Allen P, Pilar M, Walsh-Bailey C, Hooley C, Mazzucca S, Lewis CC, et al. Quantitative measures of health policy implementation determinants and outcomes: a systematic review. Implement Sci. 2020;15(1):47. | Not a knowledge communication/dissemination strategy |
| 1. Almeda N, García-Alonso CR, Salinas-Pérez JA, Gutiérrez-Colosía MR, Salvador-Carulla L. Causal Modelling for Supporting Planning and Management of Mental Health Services and Systems: A Systematic Review. Int J Environ Res Public Health. 2019;16(3). | Not a knowledge communication/dissemination strategy |
| 1. Ara R, Basarir H, Keetharuth AD, Barbieri M, Weatherly HL, Sculpher MJ, et al. Are policy decisions on surgical procedures informed by robust economic evidence? A systematic review. Int J Technol Assess Health Care. 2014;30(4):381-93. | Not a knowledge communication/dissemination strategy |
| 1. Armstrong R, Waters E, Dobbins M, Anderson L, Moore L, Petticrew M, et al. Knowledge translation strategies to improve the use of evidence in public health decision making in local government: intervention design and implementation plan. Implementation science : IS. 2013;8(1):121. | Not a systematic review |
| 1. Armstrong R, Waters E, Dobbins M, Lavis JN, Petticrew M, Christensen R. Knowledge translation strategies for facilitating evidence-informed public health decision making among managers and policy-makers. Cochrane Database of Systematic Reviews. 2011;(6):CD009181. doi:10.1002/14651858. CD009181. | Protocol |
| 1. Ashcraft LE, Quinn DA, Brownson RC. Strategies for effective dissemination of research to United States policymakers: a systematic review. Implement Sci. 2020;15(1):89. | Not a systematic review of effects/impact |
| 1. Barac R, Stein S, Bruce B, Barwick M: Scoping review of toolkits as a knowledge translation strategy in health. BMC Medical Informatics and Decision Making 2014, 14(1):1-9. | Not effectiveness measures |
| 1. Bennett K, Rhodes AE, Duda S, Cheung AH, Manassis K, Links P, et al. A Youth Suicide Prevention Plan for Canada: A Systematic Review of Reviews. Can J Psychiatry. 2015;60(6):245-57. | Not a systematic review of effects/impact |
| 1. Bergeron K, Abdi S, DeCorby K, Mensah G, Rempel B, Manson H. Theories, models and frameworks used in capacity building interventions relevant to public health: a systematic review. BMC Public Health. 2017 Nov 28;17(1):914. doi: 10.1186/s12889-017-4919-y. PMID: 29183296; PMCID: PMC5706342. | Frameworks |
| 1. Bornstein S, Baker R, Navarro P, Mackey S, Speed D, Sullivan M. Putting research in place: an innovative approach to providing contextualized evidence synthesis for decision makers. Syst Rev. 2017;6(1):218. | Not a systematic review of effects/impact |
| 1. Contandriopoulos D, Lemire M, Denis JL, Tremblay E. Knowledge exchange processes in organizations and policy arenas: a narrative systematic review of the literature. Milbank Q. 2010;88(4):444-83. | Not a systematic review |
| 1. Dagenais C, Malo M, Robert E, Ouimet M, Berthelette D, Ridde V: Knowledge transfer on complex social interventions in public health: a scoping study. PloS one 2013, 8(12):e80233. | Not effectiveness measures |
| 1. Edwards A, Zweigenthal V, Olivier J. Evidence map of knowledge translation strategies, outcomes, facilitators and barriers in African health systems. Health Res Policy Syst. 2019;17(1):16. | Not a systematic review of effects/impact |
| 1. Esmail, R., Hanson, H., Holroyd-Leduc, J. et al. A scoping review of full-spectrum knowledge translation theories, models, and frameworks. Implementation Sci 15, 11 (2020). https://doi.org/10.1186/s13012-020-0964-5 | Not effectiveness measures |
| 1. Fagerlin A, Ubel PA, Smith DM, Zikmund-Fisher BJ. Making numbers matter: present and future research in risk communication. Am J Health Behav. 2007 Sep-Oct;31 Suppl 1:S47-56. doi: 10.5555/ajhb.2007.31.supp.S47. PMID: 17931136. https://pubmed.ncbi.nlm.nih.gov/17931136/ | Not a systematic review of effects/impact |
| 1. Farrer L, Marinetti C, Cavaco YK, Costongs C. Advocacy for health equity: a synthesis review. Milbank Q. 2015;93(2):392-437. | Not a knowledge communication/dissemination strategy |
| 1. Flodgren G, Pomey MP, Taber SA, Eccles MP. Effectiveness of external inspection of compliance with standards in improving healthcare organisation behaviour, healthcare professional behaviour or patient outcomes. Cochrane Database Syst Rev. 2011:CD008992 | Not effectiveness measures |
| 1. Gifford WA, Squires JE, Angus DE, Ashley LA, Brosseau L, Craik JM, et al. Managerial leadership for research use in nursing and allied health care professions: a systematic review. Implement Sci. 2018;13(1):127. | Not targeted at policymakers or managers |
| 1. Greenhalgh C, Montgomery P. A systematic review of the barriers to and facilitators of the use of evidence by philanthropists when determining which charities (including health charities or programmes) to fund. Syst Rev. 2020;9(1):199. | Not a systematic review of effects/impact |
| 1. Hayden JA, Killian L, Zygmunt A, Babineau J, Martin-Misener R, Jensen JL, et al. Methods of a multi-faceted rapid knowledge synthesis project to inform the implementation of a new health service model: Collaborative Emergency Centres. Syst Rev. 2015;4(1):7. | Not a knowledge communication/dissemination strategy |
| 1. Horsley T, Hyde C, Santesso N, Parkes J, Milne R, Stewart R. Teaching critical appraisal skills in healthcare settings. Cochrane Database of Systematic Reviews 2011, Issue 11. Art. No.: CD001270. DOI: 10.1002/14651858.CD001270.pub2. | Not targeted at policymakers or managers |
| 1. Humphries S, Stafinski T, Mumtaz Z, Menon D. Barriers and facilitators to evidence-use in program management: a systematic review of the literature. BMC Health Serv Res. 2014;14:171. | Not a systematic review of effects/impact |
| 1. Jakobsen MW, Eklund Karlsson L, Skovgaard T, Aro AR. Organisational factors that facilitate research use in public health policy-making: A scoping review. Health Research Policy and Systems. 2019;17(1). | Not a systematic review of effects/impact |
| 1. Jones CA, Roop SC, Pohar SL, Albrecht L, Scott SD. Knowledge Translation and Implementation Special Series. Translating Knowledge in Rehabilitation: Systematic Review. Physical Therapy. 2015;95(4):663-77. | Not targeted at policymakers or managers |
| 1. Kakemam E, Liang Z, Janati A, Arab-Zozani M, Mohaghegh B, Gholizadeh M. Leadership and Management Competencies for Hospital Managers: A Systematic | Not a knowledge communication/dissemination strategy |
| 1. Kapiriri L, Razavi D. How have systematic priority setting approaches influenced policy making? A synthesis of the current literature. Health Policy. 2017;121(9):937-46. | Not a knowledge communication/dissemination strategy |
| 1. Karamitri I, Talias MA, Bellali T. Knowledge management practices in healthcare settings: a systematic review. Int J Health Plann Manage. 2017;32(1):4-18. | Not targeted at policymakers or managers |
| 1. Khalid AF, Lavis JN, El-Jardali F, Vanstone M. Supporting the use of research evidence in decision-making in crisis zones in Low- And middle-income countries: A critical interpretive synthesis. Health Research Policy and Systems. 2020;18(1). | Not a systematic review of effects/impact |
| 1. Kneale D, Rojas-García A, Raine R, Thomas J. The use of evidence in English local public health decision-making: a systematic scoping review. Implement Sci. 2017;12(1):53. | Not a systematic review of effects/impact |
| 1. Koon AD, Windmeyer L, Bigdeli M, Charles J, El Jardali F, Uneke J, et al. A scoping review of the uses and institutionalisation of knowledge for health policy in Low- and middle-income countries. Health Res Policy Syst. 2020;18(1):7. | Not effectiveness measures |
| 1. Lawrence LM, Bishop A, Curran J. Integrated Knowledge Translation with Public Health Policy Makers: A Scoping Review. Healthcare Policy. 2019;14(3):55-77. | Not a systematic review of effects/impact |
| 1. Leaman J, Richards AA, Emslie L, O'Moore EJ. Improving health in prisons - from evidence to policy to implementation - experiences from the UK. Int J Prison Health. 2017;13(3-4):139-67. | Not targeted at policymakers or managers |
| 1. Leon N, Balakrishna Y, Hohlfeld A, Odendaal WA, Schmidt BM, Zweigenthal V, et al. Routine Health Information System (RHIS) improvements for strengthened health system management. Cochrane Database of Systematic Reviews. 2020(8). | Not a knowledge communication/dissemination strategy |
| 1. Liverani M, Hawkins B, Parkhurst JO. Political and institutional influences on the use of evidence in public health policy. A systematic review. PLoS One. 2013;8(10):e77404. | Not a knowledge communication/dissemination strategy |
| 1. Long JC, Cunningham FC, Braithwaite J. Bridges, brokers and boundary spanners in collaborative networks: a systematic review. BMC Health Serv Res. 2013;13:158. | Not a systematic review of effects/impact |
| 1. Lunden A, Teräs M, Kvist T, Häggman-Laitila A. A systematic review of factors influencing knowledge management and the nurse leaders' role. J Nurs Manag. 2017;25(6):407-20. | Not targeted at policymakers or managers |
| 1. Majid U, Kandasamy S. The rationales for and challenges with employing arts-based health services research (ABHSR): a qualitative systematic review of primary studies. Med Humanit. 2020. | Not a systematic review of effects/impact |
| 1. Masood, S., Kothari, A., & Regan, S. (2020). The use of research in public health policy: a systematic review. Evidence & Policy: A Journal of Research, Debate and Practice, 16(1), 7-43. | Not a knowledge communication/dissemination strategy |
| 1. McCormack B, Rycroft-Malone J, DeCorby K, Hutchinson AM, Bucknall T, Kent B, Schultz A, Snelgrove-Clarke E, Stetler C, Titler M: A realist review of interventions and strategies to promote evidence-informed healthcare: a focus on change agency. Implement Sci 2013, 8(1):107. | Not targeted at policymakers or managers |
| 1. McCormack L, Sheridan S, Lewis M, et al. Communication and dissemination strategies to facilitate the use of health-related evidence. 2013. In: Database of Abstracts of Reviews of Effects (DARE): Quality-assessed Reviews [Internet]. York (UK): Centre for Reviews and Dissemination (UK); 1995-. Available from: https://www.ncbi.nlm.nih.gov/books/NBK174174/ | Not targeted at policymakers or managers |
| 1. Meurk C, Leung J, Hall W, Head BW, Whiteford H. Establishing and Governing e-Mental Health Care in Australia: A Systematic Review of Challenges and A Call For Policy-Focussed Research. J Med Internet Res. 2016;18(1):e10. | Not a knowledge communication/dissemination strategy |
| 1. Milat AJ, Bauman A, Redman S: Narrative review of models and success factors for scaling up public health interventions. Implementation Science 2015, 10(1):1-11. | Not effectiveness measures |
| 1. Moat KA, Lavis JN, Abelson J. How contexts and issues influence the use of policy-relevant research syntheses: a critical interpretive synthesis. Milbank Q. 2013;91(3):604-48. | Not a systematic review of effects/impact |
| 1. Moore, G., Todd, A., & Redman, S. Strategies to increase the use of evidence from research in population health policy and programs: a rapid review. November 2009. Available from the Centre for Epidemiology and Research, NSW Department of Health. | SR updated in the included Moore 2011 |
| 1. Moullin JC, Sabater-Hernández D, Fernandez-Llimos F, Benrimoj si. A systematic review of implementation frameworks of innovations in healthcare and resulting generic implementation framework. Health Res Policy Syst. 2015;13:16. | Frameworks |
| 1. Oliver K, Innvar S, Lorenc T, Woodman J, Thomas J. A systematic review of barriers to and facilitators of the use of evidence by policymakers. BMC Health Serv Res. 2014;14:2. | Not a systematic review of effects/impact |
| 1. Oliver K, Lorenc T, Innvær S: New directions in evidence-based policy research: a critical analysis of the literature. Health Res Policy Syst 2014, 12(1):34. | Not effectiveness measures |
| 1. Orton L, Lloyd-Williams F, Taylor-Robinson D, O'Flaherty M, Capewell S. The use of research evidence in public health decision making processes: systematic review. PLoS One. 2011;6(7):e21704. | Old version |
| 1. Poot CC, van der Kleij RM, Brakema EA, Vermond D, Williams S, Cragg L, et al. From research to evidence-informed decision making: a systematic approach. Journal of Public Health. 2018;40:i3-i12. | Not a systematic review |
| 1. Punton M. How can capacity development promote evidence-informed policy making? Literature review for the Building Capacity to Use Research Evidence (BCURE) Programme. Itad, 2016 | Not a knowledge communication/dissemination strategy |
| 1. Robertson AR, Nurmatov U, Sood HS, Cresswell K, Smith P, Sheikh A. A systematic scoping review of the domains and innovations in secondary uses of digitised health-related data. J Innov Health Inform. 2016 Nov 10;23(3):611-619. doi: 10.14236/jhi.v23i3.841. PMID: 28059695. | Not a knowledge communication/dissemination strategy |
| 1. Rogers Van Katwyk S, Grimshaw JM, Nkangu M, Mendelson M, Taljaard M, Hoffman SJ. Study reporting quality among interventions to reduce antibiotic use is a barrier to evidence-informed policymaking on antimicrobial resistance: systematic review. J Antimicrob Chemother. 2020;75(5):1091-8. | Not a knowledge communication/dissemination strategy |
| 1. Rogers Van Katwyk S, Grimshaw JM, Nkangu M, Nagi R, Mendelson M, Taljaard M, et al. Government policy interventions to reduce human antimicrobial use: A systematic review and evidence map. PLoS Med. 2019;16(6):e1002819. | Not a knowledge communication/dissemination strategy |
| 1. Savoia E, Lin L, Short S, Jha A, Argentini, Klein N et. al. Evidence syntheses to support the WHO Guidelines on Emergency Risk Communication. Boston, MA: Harvard T.H. Chan School of Public Health; 2016 (http://www.who.int/risk-communication/guidance/process/Harvard-WHO-report-q1.pdf?ua=1, | Not targeted at policymakers or managers |
| 1. Scarlett J, Forsberg BC, Biermann O, Kuchenmüller T, El-Khatib Z. Indicators to evaluate organisational knowledge brokers: A scoping review. Health Research Policy and Systems. 2020;18(1). | Not a systematic review of effects/impact |
| 1. Scott MD, McQueen S, Richardson L. Teaching Health Advocacy: A Systematic Review of Educational Interventions for Postgraduate Medical Trainees. Acad Med. 2020;95(4):644-56. | Not targeted at policymakers or managers |
| 1. Sousa MJ, Pesqueira AM, Lemos C, Sousa M, Rocha Á. Decision-Making based on Big Data Analytics for People Management in Healthcare Organizations. J Med Syst. 2019;43(9):290. | Not a knowledge communication/dissemination strategy |
| 1. Sungkar Y, Considine J, Hutchinson A. Implementation of guidelines for sepsis management in emergency departments: A systematic review. Australas Emerg Care. 2018;21(4):111-20. | Not targeted at policymakers or managers |
| 1. Thompson MR, Schwartz Barcott D. The Role of the Nurse Scientist as a Knowledge Broker. J Nurs Scholarsh. 2019;51(1):26-39. | Not a systematic review of effects/impact |
| 1. Toppenberg-Pejcic D, Nosi J, Allen T, Alexander N, Vanderford M, Gamhewage G et al. Executive summary: rapid grey literature evidence review to support the Guideline on Emergency Risk Communication. Geneva: World Health Organization; 2016 (http://www.who.int/risk-communication/guidance/process/Final-Report-Executive-Summary-Rapid-Grey-Lit-Review.pdf?ua=1https://www.who.int/risk-communication/guidance/process/Final-Report-Rapid-Grey-Lit-Review.pdf | Not targeted at policymakers or managers |
| 1. Tricco AC, Ashoor HM, Cardoso R, MacDonald H, Cogo E, Kastner M, et al. Sustainability of knowledge translation interventions in healthcare decision-making: a scoping review. Implement Sci. 2016;11:55. | Not targeted at policymakers or managers |
| 1. Tricco AC, Cardoso R, Thomas SM, Motiwala S, Sullivan S, Kealey MR, et al. Barriers and facilitators to uptake of systematic reviews by policy makers and health care managers: a scoping review. Implement Sci. 2016;11:4. | Not a systematic review of effects/impact |
| 1. Tso P, Culyer AJ, Brouwers M, Dobrow MJ. Developing a decision aid to guide public sector health policy decisions: a study protocol. Implement Sci. 2011;6:46. | Not a systematic review of effects/impact |
| 1. Turner S, D'Lima D, Hudson E, Morris S, Sheringham J, Swart N, Fulop NJ. Evidence use in decision-making on introducing innovations: a systematic scoping review with stakeholder feedback. Implement Sci. 2017 Dec 4;12(1):145. doi: 10.1186/s13012-017-0669-6. PMID: 29202772; PMCID: PMC5715650. | Not a systematic review of effects/impact |
| 1. Van Spall HGC, Lee SF, Xie F, Ko DT, Thabane L, Ibrahim Q, et al. Knowledge to action: Rationale and design of the Patient-Centered Care Transitions in Heart Failure (PACT-HF) stepped wedge cluster randomized trial. Am Heart J. 2018;199:75-82. | Not a systematic review |
| 1. Verboom B, Baumann A. Mapping the Qualitative Evidence Base on the Use of Research Evidence in Health Policy-Making: A Systematic Review. Int J Health Policy Manag. 2020. | Not a systematic review of effects/impact |
| 1. Verboom B, Montgomery P, Bennett S. What factors affect evidence-informed policymaking in public health? Protocol for a systematic review of qualitative evidence using thematic synthesis. Systematic Reviews. 2016;5(1). | Protocol |
| 1. Vis C, Bührmann L, Riper H, Ossebaard HC. Health technology assessment frameworks for eHealth: A systematic review. Int J Technol Assess Health Care. 2020;36(3):204-16. | Not a systematic review of effects/impact |
| 1. Votruba N, Ziemann A, Grant J, Thornicroft G. A systematic review of frameworks for the interrelationships of mental health evidence and policy in Low- and middle-income countries. Health Res Policy Syst. 2018;16(1):85. | Not a systematic review of effects/impact |
| 1. Xiu-xia L, Ya Z, Yao-long C, Ke-hu Y, Zong-jiu Z. The reporting characteristics and methodological quality of Cochrane reviews about health policy research. Health Policy. 2015;119(4):503-10. | Not a knowledge communication/dissemination strategy |
| 1. Yadee J, Bangpan M, Thavorn K, Welch V, Tugwell P, Chaiyakunapruk N. Assessing evidence of interventions addressing inequity among migrant populations: a two-stage systematic review. Int J Equity Health. 2019;18(1):64. | Not a knowledge communication/dissemination strategy |
| 1. Yost J, Ganann R, Thompson D, ALoweni F, Newman K, Hazzan A, et al. The effectiveness of knowledge translation interventions for promoting evidence-informed decision-making among nurses in tertiary care: a systematic review and meta-analysis. Implement Sci. 2015;10:98. | Not targeted at policymakers or managers |
| 1. Zhao N, Koch-Weser S, Lischko A, Chung M. Knowledge translation strategies designed for public health decision-making settings: a scoping review. International journal of public health. 2020;65(9):1571-80. | Not a systematic review of effects/impact |
