# Supplementary material for: Assessing the impact of knowledge communication and dissemination strategies targeted at health policy-makers and managers: an overview of systematic reviews
Source: Health Res Policy Syst. 2021 Dec 6;19:140. doi: 10.1186/s12961-021-00780-4 (PMC8645346; doi:10.1186/s12961-021-00780-4)
Supplement: Supplementary file 3 — Additional file 3. Characteristics of included studies. [file 12961_2021_780_MOESM3_ESM.docx]

**Additional file 3: Characteristics of included studies**

| Last name, year. AMSTAR 2 | Objetive of the SR | Date of most recent search | Number of studies and design (for the analysis) | Country/ies | Setting | Type of decision maker | Domain (Communication, diffusion, dissemination) |
| --- | --- | --- | --- | --- | --- | --- | --- |
| Akl 2011 (1)  High | To evaluate the effects of attribute (positive versus negative) framing and of goal (gain versus loss) framing of the same health information, on understanding, perception of effectiveness, persuasiveness, and behavior of providers, policy makers, and consumers. | 2007 | 35 studies and 51 comparisons that included: Randomized controlled trials (44), quasi-randomized controlled trials (8),and cross-over study (1) | Not reported | Academic settings, community, low income neighborhoods, genetic counselling services, health clinics, ambulatory services and cardiac clinics. | Health professionals, policy makers, and consumers. | Comunnication |
| Armstrong 2011(2)  Moderate | To determine the effectiveness of strategies aimed at facilitating evidence-informed public health decision-making through the implementation of KT strategies. | 2009-2010? | 1 cluster RCT, 6 uncontrolled studies | Canada | Provincial level | Public health officers | Dissemination |
| Ball 2021(3)  Critically Low | To evaluate approaches to public engagement with research that use the arts to facilitate engagement | May 2020 | 54 sources=40 publications (original research studies), 4 reviews; the remaining ten publications were grey literature and include three reports, three blog posts and four other web-based resources including web-based guidance, a summary of an academic article. A news article and a web-based case study. The sources included qualitative or quantitative designs. None RCT were identified. Just 5 studies included policymakers. | UK, Canada, Australia, US, Taiwan | Context of the UK or other High-income country | Any adult stakeholder (including but not limited to the general public, patients, careers, healthcare staff, policymakers and funders) | All |
| Bornbaum 2015 (4)  Low | To identify and examine the activities and tasks which comprised the KB role in health-related settings and assess whether KBs have effectively contributed to KT in health-related settings | November 2014 | 29 articles (22 studies); 8 studies assessing effectiveness of KB (only 2 reliable). There were a subset of studies (n = 8) that reported evidence of changes in knowledge (n = 5), skills (n = 2), and policies or practices (n = 6) related to their KB strategies | England (6), Canada (9), Fiji (1), Scotland (1), Australia (3), France (1), Netherlands (1) | Clinical, academic, policymaking | Multiple. Studies assessing KB effectiveness for non-clinical decision making was targeted at public health officials | Dissemination |
| Brown 2020 (5)  Low | To examine the effect of digital TEKT strategies in (1) improving the capacity for evidence-based decision making by public health policy makers and practitioners, (2) changing public health policy or practice, and (3) changes in individual or population health outcomes. | October 2018 | 8 studies (RCTs) | US (3), the Netherlands (2), Canada (2), China (1) | Healthcare services, schools, community agencies, and policy-making bodies | Health professionals ( nurses, nurse practitioners, physicians, child care health consultants, physiotherapists, primary health care workers, and public health practitioners). Further, program managers or coordinators of public health departments and professionals. | Dissemination-diffusion |
| Bunn 2011 (6)  Critically Low | To identify and evaluate potential strategies for increasing the impact of systematic reviews on policy. | January 2011 | 11 studies (13 papers): 1 RCT, 4 Surveys  2 Qualitative, 4 Descriptive papers | Canada (7)  Iran (1)  UK (2 )  USA (1) | Public health departments or units | Decision makers | All |
| Campbell 2018 (7)  Critically Low | To research strategies and factors likely to influence the use of research in population health policy and program delivery | July 2015 | 14 studies over 304 evaluating strategies to increase the use of research in policy and programs. | Australia | Not clear. Government human service agencies | Policymakers and managers | Communication & Dissemination |
| Chambers 2011 (8)  Critically Low | To evaluate Knowledge-translation resources | October 2009 | 7 evaluation studies:  4 Surveys of users  1 Interviews in development stage  2 Descriptives | 3 UK (3)  Australia(1)  Canada(1)  South Africa(1)  Low and middle-income countries (1) | National or local government or health authorities | Healthcare policy makers at the national or local level | Dissemination |
| Christine(9)  Low | To assess the effects of interventions to improve the uptake of research into health policies in low and middle-income countries | 2010 | 25 studies (1 RCT, 24 case studies) | Kenya, Ghana, Tanzania, Uganda, West African countries, Zambia (2), Bangladesh, Cambodia, Nepal, Vietnam (2), Nigeria, South Africa (2), China (2), Iran, Philippines, Brazil, Guatemala, Mexico | 12 of the studies dealt with a broad area of health care (e.g. general health service improvement, health insurance coverage, health promotion), 13 dealt with specific medical areas (e.g. a range of studies of maternal and child health, issues like mental health, malaria, etc.). | Policymakers and researchers | Dissemination |
| Dodd 2019 (10)  Critically Low | Critical factors that have facilitated the diffusion of scientific evidence into multiple phases of health policymaking in Bangladesh | 2017 | 24 studies identified (16 qualitative and 8 mixed methods) | Bangladesh | Health policy issues population not reported | High level policy-maker, directors, and managers | All |
| Fadlallah 2019(11)  High | To synthetize the evidence on the use of narratives to impact health policy-making. | February 2017 | 18 studies (15 case studies, 1 participatory action research, 1 documentary analysis, 1 biographical methods) | Australia (4), Republic of Ireland (1), US (8), UK (1), Georgia (1), China (1), South Africa (2), Rwanda (1), Republic Ireland/Australia (1) | Multiple | Policymakers (8), national healthcare organizations (1), multiple stakeholders (9) (including government and the public) | Communication |
| Haynes 2018 (12)  Critically Low | What causal mechanisms can best explain the observed outcomes of interventions that aim to increase policy-makers’ capacity to use research in their work? | 2016 | 22 studies (12 observational, 7 interventional and 3 experimental) | 18 countries: Canada (5), Australia (3), Nigeria (3), the Netherlands (2), Burkina Faso (1), Ethiopia (1), Fiji (1), USA (1), multicountry (4), multicountry collaboration (1). | The majority included bureaucrats in government departments of health or equivalent at the regional level (11 studies) or national/international level (9 studies). | Policymaker | Dissemination (the interventions are centred in increasing the capacity/ability for using evidence) |
| LaRocca 2012(13)  Critically Low | To identify the effectiveness of KT strategies used to promote evidence-informed decision making among public health decision makers. | April 2010 | 5 studies: 4 RCTs, 1 interrupted time series | Canada(2), England (1), Norway (1), USA (1) | Public health or community setting | Public health and health promotion decision makers (practitioners, managers and policy makers) | All |
| Mitton 2007 (14)  Critically Low | To examine and summarize the current evidence base for KTE in relation to health policy, resulting in an evidence-based resource for planning KTE processes | January 2006 | 10 were implementation studies (1 Post-test with control group)  2 Single group post-test  2 Multiple case study  1 Parallel case study  4 Case study or report)  ----18 studies assessing a specific KTE mechanism (but only 3 with useful designs to assess effectiveness). Additionaly, an RCT was identified in the gray literature. Designs: 2 multiple case-study studies, 1 two groups post test | Canada (8)  UK (2)  Other(4) | Public health units at the provincial level, manufacturing companies | Public health officials, occupational health decision-makers | Dissemination |
| Moore 2011(15)  Critically Low | To analyze what is known about the extent to which strategies to increase the use of research in population health policies and programs are effective | September 2009 | 5 intervention studies: 1 RCT, 1 cluster-RCT,  1 matched case control, 1 Post-intervention survey,  1 Self-assessment pre and post-intervention | 4 Canada  1 UK | Government public health department or unit; Health services | Health policy makers; program managers | Dissemination |
| Murthy 2012(16)  High | To identify and assess the effects of information products based on the findings of systematic review evidence and organizational supports and processes designed to support the uptake of systematic review evidence by health system managers, policy makers and healthcare professionals. | March 2012 | 8 studies: 3 RCTs  2 cluster RCTs  3 interrupted times series | UK (5), Canada (1), Mexico (1) , Thailand (1) | Clinical care settings, a government public health department | Health system managers, policy makers. Just one cluster RCT was addressed to public health officers | All |
| Petkovic 2016 (17)  High | To (1) assess the effectiveness of evidence summaries on policymakers’ use of the evidence and (2) identify the most effective summary components for increasing policymakers’ use of the evidence. | Not reported | Six completed RCTs (reported in seven articles) | Canada (1), Kenya (1), USA (1), unspecified countries (46% from HIC countries) (1), mixed (1), not reported (1) and two protocols. | Diverse: state-level, local-level, Legislative staff member, Health care services, public health departments, NGOs, academia, etc. | Healthcare policy-makers or managers. | Communication |
| Partridge 2010(18)  Low | (1) describe the activities and outputs of KT platforms; (2) formatively evaluate these activities and outputs; (3) summatively evaluate whether activities and outputs achieve outcomes and impacts; (4) describe the KT platforms’ context and infrastructure; and (5) examine other types of linkages among variables (which we call ‘linkage evaluations’). | 9/1/2016----July 2017? | 38 studies (17 of them included information about summative evaluations but none of them used a pre-post design or a control group) | Uganda, Cameroon  Burkina Faso, Zambia, South Africa, Thailand, Burkina Faso, Ethiopia, Nigeria, Bangladesh. | Different levels of decision-making (national, regional, provincial) | Policymakers and public health | Dissemination |
| Perrier 2011(19)  Low | To determine the impact on professional performance and healthcare outcomes of interventions for seeking, appraising, and applying evidence from systematic reviews in decision making by health policymakers | April 2010 | 2 studies (4 papers): 1 RCT  1 Post-intervention surveys at 3 months and 2 years | Canada (2) | Public Health Branch | Health policy makers and Managers | All |
| Petkovic 2016 | To assess the effectiveness of evidence summaries on policymakers’ use of the evidence and identify the most effective summary components for increasing policymakers’ use of the evidence. | Not reported | Six completed RCTs (reported in seven articles) | Canada (1), Kenya (1), USA (1), unspecified countries (1), and multiple countries (1), other study (?), and two protocols ( Canada and UK) | State-level, local-level, Legislative staff member, Health care services, public health departments, NGOs, academia, etc. | Healthcare policy-makers or managers | Disemination |
| Quinn 2014 (20)  Critically Low | Nature of knowledge exchange portals and their contribution to knowledge management in public health | December 2013 | 15 articles, including grey literature | Canada, Europe, USA | Public health units | Policy makers or practitioners in evidence-informed decision making | All |
| Sarkies 2017(21)  High | To evaluate the effectiveness of research implementation strategies for promoting evidence-informed policy and management decisions in healthcare and, to describe factors perceived to be associated with effective strategies and the inter-relationship between these factors. | February 02, 2016 | 19 studies (21 articles): two randomised controlled trials, one quasi-experimental , four program evaluations , three implementation evaluations, three mixed methods, two case studies, one survey evaluation , one pro | Australia, Burkina Faso, Canada, Fiji, Netherlands, Nigeria, multi-national, UK, USA | Healthcare organisations or government institutions. | Healthcare policy-makers or managers. | All |
| Tait 2019 (22)  Critically Low | To determine the extent of the literature on training programs designed to improve researcher competency in KT and to describe existing training methods that may be used by those hoping to build capacity for partnership research | July 2019 | 9 studies, just 3 included policymakers and researchers. Two pre-post evaluations and 1 cross-sectional study. | Canada (n = 3), Nigeria (n = 3), America (n = 1), Australia (n = 1) and England (n = 1). | Development of, curriculum for, or evaluation of KT and/or partnership research training programs | policy-makers, healthcare managers, directors of NGOs | Dissemination |
| Tate 2019(23)  Low | To determine effectiveness of interventions to enhance HCMs’ use of research in management practice | April 2016 | 7 studies (5 qulitative, 1 mixed-methods, 1 quasi-experimental) | Canada (4), US(1), Australia (1), UK (1) |  | Managers | Dissemination |
| Uneke 2017(24)  Critically Low | To assess the efforts and various initiatives that have been undertaken to deliberately engage policymakers and other stakeholders in the health sector in Nigeria for the promotion of evidence informed policymaking. | August 2015 | 14 studies (cross sectional studies, descriptive and qualitaive methods studies) | Nigeria | Diverse | Researchers and policymakers | Dissemination (10 /14 studies the intervention was training workshop, capacity building) |
| Uneke 2020(25)  Critically Low | To examine two key examples of evidence-based strategies used to successfully implement health interventions ineach of the West African countries and to highlight lessons learned, and their implication for evidence-to-policy link. | April 2020 | 30 studies | 15 West African countries |  | Policymakers | Dissemination |
| Wallace 2014(26)  High | To identify interventions to enhance evidence uptake from systematic reviews, meta-analyses and the databases containing them. | January 2014 | 10 studies:5 RCTs,  3 cluster-RCTs, 1 CCT,  1 before and after | UK (4),Australia (1), Canada(1), USA (1), Germany, Hungary,  Spain, Switzerland and the UK (1), UK and Netherlands (1), Mexico and Thailand (1) | Hospitals, a government public health department, and academic settings | All decisionmakers and policymakers | All |
| Williamson 2015(27)  Critically Low | To explore what is known about the effectiveness of strategies to increase the use of research in mental health policies. | 2013 | 9 intervention studies (2 RCT, 1 rolling cohort design, 6 case study approach) | US, UK, Canada | Entire counties or communities, teams of local authorities and agencies, and community mental health service teams | Decision makers (and brokers 1 study) | All |

References

1. Akl EA, Oxman AD, Herrin J, Vist GE, Terrenato I, Sperati F, et al. Framing of health information messages. Cochrane Database Syst Rev. 2011(12):CD006777.

2. ARMSTRONG R. Evidence-informed public health decision-making in local government 2011.

3. Ball S, Leach B, Bousfield J, Smith P, Marjanovic S. Arts-based approaches to public engagement with research: Lessons from a rapid review: RAND Corporation; 2021.

4. Bornbaum CC, Kornas K, Peirson L, Rosella LC. Exploring the function and effectiveness of knowledge brokers as facilitators of knowledge translation in health-related settings: a systematic review and thematic analysis. Implement Sci. 2015;10:162.

5. Brown A, Barnes C, Byaruhanga J, McLaughlin M, Hodder RK, Booth D, et al. Effectiveness of Technology-Enabled Knowledge Translation Strategies in Improving the Use of Research in Public Health: Systematic Review. J Med Internet Res. 2020;22(7):e17274.

6. Bunn F, Sworn K. Strategies to promote the impact of systematic reviews on healthcare policy: a systematic review of the literature. Evidence & Policy: A Journal of Research, Debate and Practice. 2011;7(4):403-28.

7. Campbell D, Moore G. Increasing the use of research in population health policies and programs: a rapid review. Public Health Research & Practice.

8. Chambers D, Wilson PM, Thompson CA, Hanbury A, Farley K, Light K. Maximizing the impact of systematic reviews in health care decision making: a systematic scoping review of knowledge-translation resources. Milbank Q. 2011;89(1):131-56.

9. Christine C, Susan C, Lisa D, Wendy G. What are the effects of interventions to improve the uptake of evidence from health research into policy in low and middle-income countries. Final report to DFID. 2011.

10. Dodd M, Ivers R, Zwi AB, Rahman A, Jagnoor J. Investigating the process of evidence-informed health policymaking in Bangladesh: a systematic review. Health Policy Plan. 2019;34(6):469-78.

11. Fadlallah R, El-Jardali F, Nomier M, Hemadi N, Arif K, Langlois EV, et al. Using narratives to impact health policy-making: A systematic review. Health Research Policy and Systems. 2019;17(1).

12. Haynes A, Rowbotham SJ, Redman S, Brennan S, Williamson A, Moore G. What can we learn from interventions that aim to increase policy-makers' capacity to use research? A realist scoping review. Federal Science Library - Canada. 2018;16(1).

13. LaRocca R, Yost J, Dobbins M, Ciliska D, Butt M. The effectiveness of knowledge translation strategies used in public health: a systematic review. BMC Public Health. 2012;12:751.

14. Mitton C, Adair CE, McKenzie E, Patten SB, Waye Perry B. Knowledge transfer and exchange: review and synthesis of the literature. Milbank Q. 2007;85(4):729-68.

15. Moore G, Redman S, Haines M, Todd A. What works to increase the use of research in population health policy and programmes: a review. Evidence & Policy: A Journal of Research, Debate and Practice. 2011;7(3):277-305.

16. Murthy L, Shepperd S, Clarke MJ, Garner SE, Lavis JN, Perrier L, et al. Interventions to improve the use of systematic reviews in decision-making by health system managers, policy makers and clinicians. Cochrane Database Syst Rev. 2012(9):Cd009401.

17. Petkovic J, Welch V, Jacob MH, Yoganathan M, Ayala AP, Cunningham H, et al. The effectiveness of evidence summaries on health policymakers and health system managers use of evidence from systematic reviews: a systematic review. Implementation Science. 2016;11:1-14.

18. Partridge ACR, Mansilla C, Randhawa H, Lavis JN, El-Jardali F, Sewankambo NK. Lessons learned from descriptions and evaluations of knowledge translation platforms supporting evidence-informed policy-making in low- and middle-income countries: a systematic review. Health Res Policy Syst. 2020;18(1):127.

19. Perrier L, Mrklas K, Lavis JN, Straus SE. Interventions encouraging the use of systematic reviews by health policymakers and managers: a systematic review. Implement Sci. 2011;6:43.

20. Quinn E, Huckel-Schneider C, Campbell D, Seale H, Milat AJ. How can knowledge exchange portals assist in knowledge management for evidence-informed decision making in public health? BMC public health. 2014;14:443.

21. Sarkies MN, Bowles KA, Skinner EH, Haas R, Lane H, Haines TP. The effectiveness of research implementation strategies for promoting evidence-informed policy and management decisions in healthcare: a systematic review. Implement Sci. 2017;12(1):132.

22. Tait H, Williamson A. A literature review of knowledge translation and partnership research training programs for health researchers. Health research policy and systems. 2019;17(1):1-14.

23. Tate K, Hewko S, McLane P, Baxter P, Perry K, Armijo-Olivo S, et al. Learning to lead: a review and synthesis of literature examining health care managers' use of knowledge. Journal of Health Services Research and Policy. 2019;24(1):57-70.

24. Uneke CJ, Sombie I, Keita N, Lokossou V, Johnson E, Ongolo-Zogo P. An assessment of policymakers' engagement initiatives to promote evidence informed health policy making in Nigeria. The Pan African medical journal. 2017;27:57.

25. Uneke CJ, Sombie I, Johnson E, Uneke BI. Lessons Learned from Strategies for Promotion of Evidence-to-Policy Process in Health Interventions in the ECOWAS Region: A Rapid Review. Nigerian medical journal : journal of the Nigeria Medical Association. 2020;61(5):227-36.

26. Wallace J, Byrne C, Clarke M. Improving the uptake of systematic reviews: a systematic review of intervention effectiveness and relevance. BMJ Open. 2014;4(10):e005834.

27. Williamson A, Makkar SR, McGrath C, Redman S. How Can the Use of Evidence in Mental Health Policy Be Increased? A Systematic Review. Psychiatric services (Washington, DC). 2015;66(8):appips201400329.
