# Supplementary material for: Assessing the impact of knowledge communication and dissemination strategies targeted at health policy-makers and managers: an overview of systematic reviews
Source: Health Res Policy Syst. 2021 Dec 6;19:140. doi: 10.1186/s12961-021-00780-4 (PMC8645346; doi:10.1186/s12961-021-00780-4)
Supplement: Supplementary file 4 — Additional file 4. Evidence rating scheme. [file 12961_2021_780_MOESM4_ESM.docx]

## Additional file 4

## Table 1: Evidence rating scheme (based on Ryan et al. 2014^[[1]](#footnote-1)^)

| **Summary statement** | **Translation** |
| --- | --- |
| Sufficient evidence | Evidence to make a decision about the effect of the intervention(s) in relation to a specific outcome(s). This includes evidence of an effect in terms of (i) benefit or (ii) harm. Statistically significant results are considered to represent sufficient evidence on which to base decisions, but a judgement of sufficient evidence is also made based on the number of studies/participants included in the analysis for a particular outcome. A rating of sufficient evidence is often based on meta-analysis producing a statistically significant pooled result that is based on a large number of included studies/participants. This judgement may also be made based on the number of studies and/or study participants showing a statistically significant result – for example (in a narrative synthesis) a result where 12 studies of a total of 14 for a specific outcome showed a statistically significant effect of an intervention would be considered to represent sufficient evidence. |
| Some evidence | Less conclusive evidence to make a decision about the effects of a particular intervention(s) in relation to a specific outcome(s). This may be based on narrative syntheses of review results. In this case, the result is qualified according to the findings of the review - for example, ‘some evidence (5 studies of 9) reported a positive effect of .....’ (This would be based on a more equivocal set of results than those obtained for ’sufficient evidence’ above. For example, while 12/14 statistically significant studies would be classed as ’sufficient evidence’, 5/9 statistically significant studies is more equivocal and would be classed as ‘some evidence’). This may also be based on a statistically significant result obtained in a small number of studies; a statistically significant result obtained from studies with a small number of participants; or a statistically significant result obtained from studies of low quality. |
| Insufficient evidence | Not enough evidence to support decisions about the effects of the intervention(s) on the basis of the included studies. This should be interpreted as ‘no evidence of effect’, rather than ’evidence of no effect’. Statistically non-significant results are considered to represent insufficient evidence. Where the number of studies is small, and/or the number of participants included in the studies is small, insufficient evidence might reflect underpowering of the included studies to be able to detect an effect of the intervention. Where the number of studies is large, and/or the number of participants included in these studies is large, ’insufficient evidence’ may reflect underlying ineffectiveness of the intervention to affect the outcomes being examined. In such cases the intervention may additionally be described as ‘generally ineffective’ in order to separate such results from those cases where insufficient evidence is used to describe results but this is based on a small number of studies and/or participants (where non-significant results may reflect underpowering of studies rather than ineffectiveness). |
| Insufficient evidence to determine | Not enough evidence to be able to determine whether an intervention is effective or not on the basis of the included studies. This statement is about reporting gaps in the evidence (i.e. where there are too few studies to be able to determine effects), rather than the situation of the summary statement above, which is about ineffectiveness (e.g. several studies reporting a statistically non-significant result). It is likely to arise when the numbers of included studies is very small. |

## Table 2. Communication strategies and approaches (Based on McCormack et al. 2013^[[2]](#footnote-2)^)

| **Type of Communication Strategy** | **Approaches to Communication** |
| --- | --- |
| **Tailoring the message**  Communication designed for an individual based on information from the individual | • Using a computerized database of messages that can be combined in response to answers to preprogrammed questions asked of an individual.  • Applying an electronic algorithm to design messages based on individual input regarding a limited number of questions.  • Trying to direct messages to individuals’ status on key theoretical determinants (knowledge, outcome expectations, normative beliefs, efficacy. or skills) of the behavior of interest.  • Incorporating recognizable aspects of participants to convey (implicitly or explicitly) that the messages are designed specifically for them. This is more than a personalized letter (e.g., “Dear Jane”).  • Providing messages to participants about their psychological or behavioral states. Individualized feedback may be provided synchronously (e.g., via a chat function, telephone, or face-to-face) or asynchronously (e.g., via email or discussion board, or mail). |
| **Targeting the message**  Communication designed for subgroups based on group membership or characteristics such as age, sex, race, cultural background, language, and other “psychographic” characteristics (e.g., a person’s attitudes about a particular subject matter) | • Manipulating language, visuals, music, or choice of behavior topic in ways that make the message more interesting, relevant, or appealing to specific subgroups. |
| **Using narratives**  Communication delivered in the form of a story, testimonial, or entertainment education | • Invoking personal stories, case studies, anecdotes, testimonials, experiential sharing (e.g., personal account of an individual’s experience in donating an organ to a sibling).  • Using entertainment education (e.g., talking about issue in a soap opera storyline) or photo novellas or graphic novels. |
| **Framing the message**  Communication that conveys the same messages in alternative ways (e.g., emphasizing either what is gained or what is lost by taking an action or making a choice) | • Creating messages that emphasize the positive consequences of compliance are referred to as *positive (gain) frame*, whereas those that stress the negative consequence of noncompliance are denoted as *negative (loss) frame*. Studies should explicitly state that the stimuli differed in terms of gain or loss frame. For example,  o Positive (gain) frame: “Get active! Enhance your health!” vs. “A lack of activity increases risk for diabetes.”  o Negative (loss) frame: “With drug X, you have a 5% chance of dying” vs. “With drug X, you have a 95% chance of surviving.” |
| **More than one of the above strategies** | • A multicomponent approach uses several communication strategies in concurrent combination or in sequence to increase understanding of the evidence or information.  • Multicomponent interventions are important to this review only to the extent that they are compared with another intervention that is different by only 1 or more aspects. |

**Table 3. Dissemination strategies and approaches (Based on McCormack et al. 2013)**

| **Type of Dissemination Strategy** | **Approaches to Dissemination** |
| --- | --- |
| **Improve reach of evidence**  Distributing evidence widely to many audiences and across many settings extends the numbers and types of recipients. | • Postal: Any information delivered to a new destination via a human carrier employed by a government-affiliated postal service or a for-profit mail or parcel delivery service such as FedExTM or UPS®.  • Electronic and digital media: Any information delivered via telephone or web-based email, text messages, or electronic programs such as personal digital assistant (PDA) resources or phone apps.  • Social media: Any information delivered via Internet-based social networking sites such as Facebook, Twitter, YouTube, myspace, foursquare, and LinkedIn. Sometimes problem- or group-specific social networks exist for professional organizations or patient subgroups; these would fall into social media as long as they have a “social” network component as described above.  • Mass media: Any information delivered via television, radio, print newspapers, print magazines, or billboards.  • Interpersonal verbal group or individual outreach: Information delivered via telephone, webinar, or in-person visits, including purposeful delivery of brochures or pamphlets, but without any motivational component. The audiences can include: pharmacists, nurses, doctors, counselors, or other clinicians. |
| **Motivate recipients to use and apply evidence** Using a variety of authoritative experts or spokespersons to increasing interest in or acceptability of the evidence or related recommendations may promote enthusiasm or action on the part of clinicians or patients. | • Champions (cheerleaders): People who take ownership of the evidence and visibly promotes it within their own organization or across other settings. Champions help overcome social and political pressures imposed by an organization, provide a role model for personal commitment to the program, and involve others in its use.  o For example, an evidence developer might train or enlist the help of a local champion to promote evidence within his or her organization.  • Opinion or thought leaders (frequently has an endorsing or persuasive element): Recognized expertswho lend their name to dissemination efforts to endorse the idea being disseminated and to establish credibility. They may or may not actually participate in the work and do not necessarily have any relationship with the organization to which evidence is to be disseminated. They could endorse the intervention, have a role in its development, or advise on strategies.  o For example, an opinion leader might be the CEO or the head of a department, an external expert in a particular field applicable to the evidence, or a well-recognized figure such as the U.S. Surgeon General.  • Social networks: A network of individuals who have a common perspective, relationships, or similar connection. The relationships can be informal (friends, peers, family) or formal (patient provider, nurses), but network members have defined role obligations. Peer networks provide a central and trusted source for information and might use multiple other dissemination strategies themselves (such as newsletters, journals, phone- and internet-based distribution, face-to-face conferences, peer-to- peer conversations, etc.). |
| **Enhance recipients’ ability to use and apply evidence (regardless of delivery mode)**  Providing additional resources about evidence or recommendations based on evidence, such as how they can be incorporated into current practice, or giving specific suggestions for change enhances a traditional dissemination strategy. | • Provision of supporting “how-to” materials: Includes physical materials that a health care practice might use to apply evidence in their activities. These might include giving tracking sheets to patients or giving risk calculators to clinicians. These might also include tailored toolkits that explain how to implement evidence-based recommendations from in specific settings.  • Supporting materials *do not* include brochures, counseling resources, or resources that originate from the practice. They must originate from the evidence developer and be given to the end user.  • Skill training, capacity building, and problem solving: Training in any skill that would allow appropriate use of evidence (to overcome barriers); might include training in recognizing the quality of evidence or the circumstances under which it can be reasonably used; also includes training in various counseling techniques that would facilitate evidence implementation and interactive seminars. |
| **More than one of the above strategies** Combining multiple dissemination strategies, including ways to increase reach, motivation, or ability, may be more effective than single strategies. | • A multicomponent approach uses several dissemination strategies in concurrent combination or in sequence to increase the reach of evidence, enhance the end users’ motivation to adopt and use or apply evidence. Multicomponent interventions are important to this review only to the extent that they are compared with another intervention that is different by at least one other aspect. |

1. Ryan R, Santesso N, Lowe D, Hill S, Grimshaw J, Prictor M, et al. Interventions to improve safe and effective medicines use by consumers: An overview of systematic reviews. Cochrane Database of Systematic Reviews. 2014;4:CD007768. [↑](#footnote-ref-1)
2. McCormack L, Sheridan S, Lewis M, Boudewyns V, Melvin CL, Kistler C, Lux LJ, Cullen K, Lohr KN. Communication and Dissemination Strategies To Facilitate the Use of Health-Related Evidence. Evidence Report/Technology Assessment No. 213. (Prepared by the RTI International–University of North Carolina Evidence-based Practice Center under Contract No. 290-2007-10056-I.) AHRQ Publication No. 13(14)-E003-EF. Rockville, MD: Agency for Healthcare Research and Quality; November 2013. www.effectivehealthcare.ahrq.gov/reports/final.cfm. DOI: https://doi.org/10.23970/ AHRQEPCERTA213 [↑](#footnote-ref-2)
