# Supplementary material for: Assessing the impact of knowledge communication and dissemination strategies targeted at health policy-makers and managers: an overview of systematic reviews
Source: Health Res Policy Syst. 2021 Dec 6;19:140. doi: 10.1186/s12961-021-00780-4 (PMC8645346; doi:10.1186/s12961-021-00780-4)
Supplement: Supplementary file 5 — Additional file 5. AMSTAR 2 quality assessment. [file 12961_2021_780_MOESM5_ESM.docx]

**Additional file 5: AMSTAR 2 quality assessment**

| Last name, year | Q1 | Q2 | Q3 | Q4 | Q5 | Q6 | Q7 | Q8 | Q9 | Q10 | Q11 | Q12 | Q13 | Q14 | Q15 | Q16 | Score |
| --- | --- | --- | --- | --- | --- | --- | --- | --- | --- | --- | --- | --- | --- | --- | --- | --- | --- |
| Akl 2011(1) | YES | YES | YES | YES | YES | YES | YES | YES | YES | NO | NA | NA | YES | YES | YES | YES | High |
| Armstrong 2011(2) | YES | YES | YES | YES | NO | NO | YES | YES | YES | NO | NA | NA | YES | YES | NA | YES | Moderate |
| Ball 2021(3) | YES | NO | YES | YES | YES | YES | NO | YES | NA | NO | NA | NA | NO | NO | NA | YES | Critically Low |
| Bornbaum 2015 (4) | YES | P/YES | YES | YES | YES | YES | NO | YES | YES | NO | NA | NA | YES | YES | NA | YES | Low |
| Brown 2020 (5) | YES | YES | NO | YES | YES | YES | NO | YES | YES | NO | NA | NA | YES | YES | NA | YES | Low |
| Bunn 2011 (6) | YES | NO | YES | YES | YES | YES | NO | YES | YES | NO | NA | NA | YES | NA | NA | NO | Critically Low |
| Campbell 2018 (7) | YES | NO | YES | P/YES | YES | NO | NO | YES | P/YES | NO | NA | NA | NO | NA | NA | NO | Critically Low |
| Chambers 2011 (8) | YES | YES | YES | YES | NO | NO | NO | YES | NO | NO | NA | NA | NO | NA | NA | YES | Critically Low |
| Christine(9) | YES | NO | NO | YES | YES | YES | YES | YES | YES | NO | NA | NA | YES | NO | NA | YES | Low |
| Dodd 2019 (10) | YES | YES | YES | YES | YES | YES | NO | YES | YES | P/YES | NA | NA | NO | NA | NA | YES | Critically Low |
| Fadlallah 2019(11) | YES | YES | YES | YES | YES | YES | YES | YES | YES | NO | NA | NA | YES | NA | NA | YES | High |
| Haynes 2018 (12) | YES | NO | YES | P/YES | NO | NO | NO | YES | NO | NO | NA | NA | NO | NA | NA | YES | Critically Low |
| LaRocca 2012(13) | YES | NO | YES | YES | YES | YES | NO | YES | YES | NO | NA | NA | YES | NA | NA | YES | Critically Low |
| Mitton 2007 (14) | NO | NO | NO | YES | NO | NO | NO | YES | YES | NO | NA | NA | YES | NO | NA | YES | Critically Low |
| Moore 2011(15) | NO | NO | NO | NO | NO | NO | NO | YES | NO | NO | NA | NA | NO | NA | NA | YES | Critically Low |
| Murthy 2012(16) | YES | YES | YES | YES | YES | YES | YES | YES | YES | NO | NA | NA | YES | YES | NA | YES | High |
| Partridge 2010(17) | YES | NO | YES | YES | YES | YES | P/YES | YES | YES | NO | NA | NA | YES | NA | NA | YES | Low |
| Perrier 2011(18) | YES | YES | YES | YES | YES | YES | NO | YES | YES | NO | NA | NA | YES | NA | NA | YES | Low |
| Petkovic 2016(19) | YES | YES | YES | YES | YES | YES | YES | YES | YES | NO | NA | YES | YES | NA | NA | YES | High |
| Quinn 2014 (20) | YES | NO | YES | YES | YES | YES | NO | NO | NO | NO | NA | NA | NO | NA | NA | YES | Critically Low |
| Sarkies 2017(21) | YES | YES | YES | YES | YES | YES | YES | YES | YES | NO | NA | NA | YES | NA | NA | YES | High |
| Tait 2019 (22) | YES | NO | YES | YES | NO | NO | NO | YES | YES | NO | NA | NA | NO | NA | NA | YES | Critically Low |
| Tate 2019(23) | YES | YES | NO | YES | YES | YES | NO | YES | YES | NO | NA | NA | YES | NA | NA | YES | Low |
| Uneke 2017(24) | NO | NO | NO | NO | NO | NO | NO | P/YES | NO | NO | NA | NA | NO | NA | NA | YES | Critically Low |
| Uneke 2020(25) | NO | NO | NO | NO | YES | NO | NO | P/YES | NO | NO | NA | NA | NO | NA | NA | YES | Critically Low |
| Wallace 2014(26) | YES | YES | YES | YES | YES | YES | YES | YES | YES | NO | NA | NA | YES | NA | NA | YES | High |
| Williamson 2015(27) | YES | NO | YES | YES | YES | YES | NO | YES | NO | NO | NA | NA | NO | NA | NA | YES | Critically Low |

[AMSTAR II](https://amstar.ca/Amstar_Checklist.php) ^[[1]](#footnote-1)^ Questions: Q 1. Did the research questions and inclusion criteria for the review include the components of PICO?; Q 2. Did the report of the review contain an explicit statement that the review methods were established prior to the conduct of the review and did the report justify any; Q 3. Did the review authors explain their selection of the study designs for inclusion in the review?; Q 4. Did the review authors use a comprehensive literature search strategy?; Q5. Did the review authors perform study selection in duplicate?; Q 6. Did the review authors perform data extraction in duplicate?; Q 7. Did the review authors provide a list of excluded studies and justify the exclusions?; Q8. Did the review authors describe the included studies in adequate detail?; Q 9. Did the review authors use a satisfactory technique for assessing the risk of bias (RoB) in individual studies that were included in the review?; Q 10. Did the review authors report on the sources of funding for the studies included in the review?; Q11. If meta-analysis was performed, did the review authors use appropriate methods for statistical combination of results?; Q12. If meta-analysis was performed, did the review authors assess the potential impact of RoB in individual studies on the results of the meta-analysis or other evidence synthesis?; Q 13. Did the review authors account for RoB in primary studies when interpreting/discussing the results of the review?; Q14. Did the review authors provide a satisfactory explanation for, and discussion of, any heterogeneity observed in the results of the review?; Q15. If they performed quantitative synthesis did the review authors carry out an adequate investigation of publication bias (small study bias) and discuss its likely impact on the results of the review; Q16. Did the review authors report any potential sources of conflict of interest, including any funding they received for conducting the review?; CL = CRITICALLY LOW; NA = Not applicable. P/YES= PARTIALLY YES

References

1. Akl EA, Oxman AD, Herrin J, Vist GE, Terrenato I, Sperati F, et al. Framing of health information messages. Cochrane Database Syst Rev. 2011(12):CD006777.

2. ARMSTRONG R. Evidence-informed public health decision-making in local government 2011.

3. Ball S, Leach B, Bousfield J, Smith P, Marjanovic S. Arts-based approaches to public engagement with research: Lessons from a rapid review: RAND Corporation; 2021.

4. Bornbaum CC, Kornas K, Peirson L, Rosella LC. Exploring the function and effectiveness of knowledge brokers as facilitators of knowledge translation in health-related settings: a systematic review and thematic analysis. Implement Sci. 2015;10:162.

5. Brown A, Barnes C, Byaruhanga J, McLaughlin M, Hodder RK, Booth D, et al. Effectiveness of Technology-Enabled Knowledge Translation Strategies in Improving the Use of Research in Public Health: Systematic Review. J Med Internet Res. 2020;22(7):e17274.

6. Bunn F, Sworn K. Strategies to promote the impact of systematic reviews on healthcare policy: a systematic review of the literature. Evidence & Policy: A Journal of Research, Debate and Practice. 2011;7(4):403-28.

7. Campbell D, Moore G. Increasing the use of research in population health policies and programs: a rapid review. Public Health Research & Practice.

8. Chambers D, Wilson PM, Thompson CA, Hanbury A, Farley K, Light K. Maximizing the impact of systematic reviews in health care decision making: a systematic scoping review of knowledge-translation resources. Milbank Q. 2011;89(1):131-56.

9. Christine C, Susan C, Lisa D, Wendy G. What are the effects of interventions to improve the uptake of evidence from health research into policy in low and middle-income countries. Final report to DFID. 2011.

10. Dodd M, Ivers R, Zwi AB, Rahman A, Jagnoor J. Investigating the process of evidence-informed health policymaking in Bangladesh: a systematic review. Health Policy Plan. 2019;34(6):469-78.

11. Fadlallah R, El-Jardali F, Nomier M, Hemadi N, Arif K, Langlois EV, et al. Using narratives to impact health policy-making: A systematic review. Health Research Policy and Systems. 2019;17(1).

12. Haynes A, Rowbotham SJ, Redman S, Brennan S, Williamson A, Moore G. What can we learn from interventions that aim to increase policy-makers' capacity to use research? A realist scoping review. Federal Science Library - Canada. 2018;16(1).

13. LaRocca R, Yost J, Dobbins M, Ciliska D, Butt M. The effectiveness of knowledge translation strategies used in public health: a systematic review. BMC Public Health. 2012;12:751.

14. Mitton C, Adair CE, McKenzie E, Patten SB, Waye Perry B. Knowledge transfer and exchange: review and synthesis of the literature. Milbank Q. 2007;85(4):729-68.

15. Moore G, Redman S, Haines M, Todd A. What works to increase the use of research in population health policy and programmes: a review. Evidence & Policy: A Journal of Research, Debate and Practice. 2011;7(3):277-305.

16. Murthy L, Shepperd S, Clarke MJ, Garner SE, Lavis JN, Perrier L, et al. Interventions to improve the use of systematic reviews in decision-making by health system managers, policy makers and clinicians. Cochrane Database Syst Rev. 2012(9):Cd009401.

17. Partridge ACR, Mansilla C, Randhawa H, Lavis JN, El-Jardali F, Sewankambo NK. Lessons learned from descriptions and evaluations of knowledge translation platforms supporting evidence-informed policy-making in low- and middle-income countries: a systematic review. Health Res Policy Syst. 2020;18(1):127.

18. Perrier L, Mrklas K, Lavis JN, Straus SE. Interventions encouraging the use of systematic reviews by health policymakers and managers: a systematic review. Implement Sci. 2011;6:43.

19. Petkovic J, Welch V, Jacob MH, Yoganathan M, Ayala AP, Cunningham H, et al. The effectiveness of evidence summaries on health policymakers and health system managers use of evidence from systematic reviews: a systematic review. Implementation Science. 2016;11:1-14.

20. Quinn E, Huckel-Schneider C, Campbell D, Seale H, Milat AJ. How can knowledge exchange portals assist in knowledge management for evidence-informed decision making in public health? BMC public health. 2014;14:443.

21. Sarkies MN, Bowles KA, Skinner EH, Haas R, Lane H, Haines TP. The effectiveness of research implementation strategies for promoting evidence-informed policy and management decisions in healthcare: a systematic review. Implement Sci. 2017;12(1):132.

22. Tait H, Williamson A. A literature review of knowledge translation and partnership research training programs for health researchers. Health research policy and systems. 2019;17(1):1-14.

23. Tate K, Hewko S, McLane P, Baxter P, Perry K, Armijo-Olivo S, et al. Learning to lead: a review and synthesis of literature examining health care managers' use of knowledge. Journal of Health Services Research and Policy. 2019;24(1):57-70.

24. Uneke CJ, Sombie I, Keita N, Lokossou V, Johnson E, Ongolo-Zogo P. An assessment of policymakers' engagement initiatives to promote evidence informed health policy making in Nigeria. The Pan African medical journal. 2017;27:57.

25. Uneke CJ, Sombie I, Johnson E, Uneke BI. Lessons Learned from Strategies for Promotion of Evidence-to-Policy Process in Health Interventions in the ECOWAS Region: A Rapid Review. Nigerian medical journal : journal of the Nigeria Medical Association. 2020;61(5):227-36.

26. Wallace J, Byrne C, Clarke M. Improving the uptake of systematic reviews: a systematic review of intervention effectiveness and relevance. BMJ Open. 2014;4(10):e005834.

27. Williamson A, Makkar SR, McGrath C, Redman S. How Can the Use of Evidence in Mental Health Policy Be Increased? A Systematic Review. Psychiatric services (Washington, DC). 2015;66(8):appips201400329.

1. Shea BJ, Reeves BC, Wells G, Thuku M, Hamel C, Moran J, Moher D, Tugwell P, Welch V, Kristjansson E, Henry DA. AMSTAR 2: a critical appraisal tool for systematic reviews that include randomised or non-randomised studies of healthcare interventions, or both. BMJ. 2017 Sep 21;358:j4008. doi: 10.1136/bmj.j4008. [↑](#footnote-ref-1)
