# Supplementary material for: Assessing the impact of knowledge communication and dissemination strategies targeted at health policy-makers and managers: an overview of systematic reviews
Source: Health Res Policy Syst. 2021 Dec 6;19:140. doi: 10.1186/s12961-021-00780-4 (PMC8645346; doi:10.1186/s12961-021-00780-4)
Supplement: Supplementary file 6 — Additional file 6. Interventions and tools. [file 12961_2021_780_MOESM6_ESM.docx]

**Additional file 6**

Table 1. Strategies assessed in included reviews

|  | Techniques to communicate evidence | | | | | | Strategies to disseminate evidence | | | |
| --- | --- | --- | --- | --- | --- | --- | --- | --- | --- | --- |
| References | Tailoring the message | Targeting the message | Using narratives and other forms of art | Framing the message (a) | Using different presentation formats (b) | Using a multicomponent approach (c ) | Increase reach of the evidence (d) | Increase people’s motivation to use and apply the evidence (e) | Increase people’s ability to use and apply the evidence (f) | Use a multipronged approach(g) |
| Akl 2011(1) |  |  |  | X |  |  |  |  |  |  |
| Armstrong 2011(2) |  | X |  |  |  |  |  | X |  |  |
| Ball 2021(3) | X | X | X |  |  | X |  |  | X |  |
| Bornbaum 2015 (4) |  |  |  |  |  |  |  | X |  | X |
| Brown 2020 (5) |  |  |  |  |  |  |  |  |  | X |
| Bunn 2011 (6) |  | X |  |  |  |  | X | X |  |  |
| Campbell 2018 | X | X |  | X |  |  | X | X | X |  |
| Chambers 2011 (7) |  |  |  |  |  |  | X |  |  |  |
| Christine 2011 (8) |  |  |  |  |  |  |  | X | X |  |
| Dodd 2019(9) |  |  |  | X |  |  | X | X | X | X |
| Fadlallah 2019(10) |  |  | X |  |  |  |  |  |  |  |
| Haynes 2018(11) |  | X |  |  |  |  | X | X | X |  |
| LaRocca 2012(12) | X | X |  |  |  |  | X | X | X |  |
| Mitton 2007 (13) |  | X |  |  |  |  | X | X |  |  |
| Moore 2011(14) | X | X |  |  |  |  |  | X |  |  |
| Murthy 2012(15) | X | X |  |  |  |  | X | X | X |  |
| Partridge 2010(16) |  |  |  |  |  |  |  |  |  | X |
| Perrier 2011(17) | X | X |  |  |  |  | X | X |  |  |
| Petkovic 2016(18) | X | X | X |  | X |  |  |  |  |  |
| Quinn 2014(19) | X | X |  |  |  |  | X | X |  |  |
| Sarkies 2017(20) | X | X |  |  |  | X | X | X | X |  |
| Tait 2019(21) |  |  |  |  |  | X | X |  | X |  |
| Tate 2019(22) |  |  |  |  |  |  |  |  | X |  |
| Uneke 2020(23) |  |  |  |  |  |  |  |  | X |  |
| Uneke 2017(24) |  |  |  |  |  |  |  |  |  | X |
| Wallace 2014(25) | X |  |  |  |  |  | X | X |  |  |
| Williamson 2015(26) |  |  |  |  |  | X | X | X | X | X |

1. (e.g., Messages that emphasize the positive consequences of compliance are referred to as a positive (gain) frame, whereas a version that stresses the negative consequence of noncompliance is called a negative (loss) frame.
2. (e.g., graphical, numeric, non-numeric)
3. (e.g., uses several communication techniques in concurrent combination or in sequence to increase the comprehension and understanding of evidence)
4. (e.g., uses several communication techniques in concurrent combination or in sequence to increase the comprehension and understanding of evidence)
5. (e.g., e-mail alerts, electronic/digital media, social media, mass media; interpersonal outreach, webinars, brochures/pamphlets).
6. (e.g., opinion leaders, champions, social networks, knowledge brokers).
7. (e.g., additional resources, skills building, it might also include tailored toolkits that explain implementation of evidence in specific settings)
8. (e.g., social marketing, academic detailing)

Table 2. Formats or tools assessed in the strategies

|  | Formats or tools | | | | | |
| --- | --- | --- | --- | --- | --- | --- |
| Reference | Physical or printed tools | Electronic | Internet based | Interpersonal | Media (newspapers, radio, tv); social media | Details |
| Akl 2011(1) | X | X |  |  |  | Video, multimedia, Booklet |
| Armstrong 2011(2) |  | X | X | X |  |  |
| Ball 2021(3) | X |  |  | X |  | Arts-based methods (visual arts, performing arts, etc) |
| Bornbaum 2015 (4) |  | X | X | X |  |  |
| Brown 2020 (5) |  | X | X |  |  |  |
| Bunn 2011 (6) |  |  |  | X |  | SRs, knowledge broker, register of synthesized and translated research evidence |
| Campbell 2018 (27) |  |  | X | X | X |  |
| Chambers 2011 (7) | X | X |  | X |  | Summaries, policy briefs, overviews |
| Christine 2011 (8) |  |  |  |  |  |  |
| Dodd 2019(9) | X |  |  | X |  | Policy briefs, policy dialogues,workshops, advocacy groups, national data |
| Fadlallah 2019(10) | X | X | X |  | X |  |
| Haynes 2018(11) |  | X | X | X |  | Platforms for ongoing interactivity, e.g. community of practice, cross-sector committees |
| LaRocca 2012(12) |  | X | X | X |  | KT strategies ( educational sessions; dissemination channels including print, CD-Rom, etc.) |
| Mitton 2007 (13) |  |  | X | X |  |  |
| Moore 2011(14) |  | X |  |  |  | SRs , tailored messaging , knowledge brokers |
| Murthy 2012(15) | X | X | X | X |  | Bulletin, systematic reviews access, |
| Partridge 2010(16) |  |  | X | X |  | Clearinghouses, Deliberative dialogues. |
| Perrier 2011(17) |  |  | X | X |  |  |
| Petkovic 2016(18) | X | X | X | X |  | All the dissemination strategies were through email, (one combined with follow up telephone call or emailed if preferred) : Printed leaflet/ booklet, PDF version for those who prefer online, Table, and Text. |
| Quinn 2014(19) |  |  | X | X | X | Wikis, blogs and forums and online training video modules, knowledge translation tools and methods, epidemiological and demographic data. |
| Sarkies 2017(20) | X | X | X | X |  |  |
| Tait 2019(21) |  | X |  | X |  | Programmes for capacity building on KT |
| Tate 2019(22) |  |  |  | X |  |  |
| Uneke 2020(23) |  |  | X | X |  |  |
| Uneke 2017(24) |  |  |  | X |  |  |
| Wallace 2014(25) |  |  | X | X |  |  |
| Williamson 2015(26) |  |  | X | X | X | Geographical Information System data and in how to use these data to inform policy and planning decisions |

Table 3. Characteristics of interventions

| Reference | Interventions |
| --- | --- |
| Akl 2011(1) | Consisted of positively versus negatively framed messages (attribute framing) or gain-framed versus loss framed messages (goal framing). The two messages being compared should describe the same health information. Were excluded studies of risk choice framing. The 51 comparisons were: 13 related to attribute framing and 38 of goal framing. The messages used in these comparisons were about: • screening (n = 19), • prevention (n = 19), • treatment (n = 8), and • other (n = 5: 2 harm, 1 diagnosis, 1 public health, 1 on abortion); 1 study used 2 types of messages. They used videos, information, messages, pamphlet, brochure, reminder letters framed, information about condition, multimedia, booklet, articles, hypothetical situations. |
| Armstrong 2011(2) | The interventions arms in the only study included were: targeted messages (TM) and TM + knowledge brokers. Website was the control arm ('usual care'). |
| Ball 2021(3) | Many arts-based engagement projects include elements of training or skills-building, for example, explaining an engagement process, research topic or arts-based intervention to stakeholders before its development or delivery. Visual arts, Games, Performing arts, Arts-based knowledge production and translation – general observations were identified as evidence support of effectiveness for policymakers. |
| Bornbaum 2015 (4) | Knowledge brokering: capacity building activities, small group workshops, one-to-one consultations, development of policy briefs, access to a repository of SR, tailored & targeted messages. |
| Brown 2020 (5) | Five of the interventions were web-based training programs aimed at improving nutrition and physical activity knowledge in children, utilizing smoking cessation guidelines, body positivity in children and adolescents, physical activity. |
| Bunn 2011 (6) | Collaborative approaches between researchers and decision makers; Collaborative approaches & SRs; Dissemination strategies. |
| Campbell 2018 (27) | Policy briefs to communicate evidence from research, Commissioning rapid reviews of research, A writing tool to improve communication of evidence from research, Seconding policymakers into research teams, Structured seminar series to promote interaction between policymakers and researchers, Conference technology to support knowledge sharing, Networks to support knowledge production and exchange, Organisation-wide capacity development initiatives, Grant-funded collaboration involving policymakers, practitioners and university department. |
| Chambers 2011 (7) | Workshops + surveys |
| Christine 2011 (8) | Diverse: Operations research (Participatory Ethnographic Evaluation and Research (PEER)), Intersectoral dialogue with key stakeholders and policymakers at the local, provincial and central level, Dissemination (dissemination forums) and advocacy, Education and capacity building, participatory implementation, etc. |
| Dodd 2019(9) | Not well described. We identified KTPs, workshops, deliberative dialogues, etc. |
| Fadlallah 2019(10) | Meta-narrative, which combined the stories of a large number of people to convey a thematic, systemic story as opposed to focusing on a single event or individual (i.e. episodic stories). The narrative information was presented in different formats, with some studies utilizing more than one format. These formats included television appearances, entertainment education (prime-time network TV storyline), short films, theatre and plays, magazines and journal print, books, narrative action reflection workshops, video materials, posters and photos, booklets with testimonies, advocacy summaries of personal experiences, micro-blogs and online forums, and verbal (narrated). |
| Haynes 2018(11) | ‘Access to research’, ‘Skills improvement’ in accessing, appraising and/or applying research in policy work,‘Systems improvement’ tackling organisational or cross-organisational infrastructure, processes and/or resources, and ‘Interaction’ with researchers. |
| LaRocca 2012(12) | Communities of practice (CoPs); Education; Education + information service + free access to databases; Dissemination of information; Access to SRs +/- tailored messaging +/- knowledge brokers. |
| Mitton 2007 (13) | Face-to-face exchange; Education; Networks and CoPs; Facilitated meetings ( decision makers and researchers); Web-based information and communication. KB (2 studies), promoting "interacting" teams (1 study), "linking systems" (1 study). All of them seems to be a kind of exchange/brokering intervention. |
| Moore 2011(14) | Access to SRs +/- tailored messaging +/- knowledge brokers; Dissemination of SRs; Interaction between users and producers of research; Education. |
| Murthy 2012(15) | [Intervention 1:Knowledge Brokers (KB) + Tailored Message (TM) + Health evidence (HE), Intervention 2: Tailored message (TM): Targeted messages plus access to health-evidence. ca (short summaries and full text), Intervention 3: control group: access to health-evidence.ca](http://health-evidence.ca/) |
| Partridge 2010(16) | The focus is on KT platforms using different types of activities and outputs |
| Perrier 2011(17) | Dissemination of SRs; website, tailored messages, knowledge broker |
| Petkovic 2016(18) | All the dissemination strategies were through email, (one combined with follow up telephone call or emailed if preferred) : Printed leaflet/ booklet, PDF version for those who prefer online, Table, and Text. =Two studies assessed policy briefs; one assessed an “evidence summary”; two assessed different formats of summary of findings tables, which are distinct table formats presenting the main findings of the review (absolute and relative effects for each important outcome) and quality of the evidence; and one compared an SOF table alone to a summary of findings table as part of a “graded entry” evidence summary (a short one-page summary, then a narrative report, followed by access to the complete systematic review). Two studies assessed evidence summaries which included recommendations for programs or policies, while the others did not specify whether recommendations were provided within the summary. |
| Quinn 2014(19) | (i) access to a KEP (HealthEvidence.ca); (ii) access to HealthEvidence.ca plus tailored electronic messaging (TM) and (iii) access to HealthEvidence.ca plus tailored messaging plus access to an organisational knowledge broker (KB). |
| Sarkies 2017(20) | Policy brief accompanied by an expert opinion piece or tailored targeted messages with addition of a knowledge broker. |
| Tait 2019(21) | All programs were: delivered face-to-face, included practical skills-building opportunities, and employed multiple learning modalities such as seminars and small group discussions. Details: Power point, handouts, lectures and interactive discussions, question/answer sessions, focus groups, policy dialogue. |
| Tate 2019(22) | Evidence-based public health training course; a computer based/desktop application; meeting-based, executive-level knowledge translation activities and a residency programme. |
| Uneke 2020(23) | Training workshops (include PBs development, priority setting excercises, deliberative dialogues, etc) |
| Uneke 2017(24) | Multiple approaches: RRS, policy dialogues, capacity strengthening, steering committees, multisectoral action and consultative processes. |
| Wallace 2014(25) | Just one study focused on decisionmakers: Messages from 7 rigorous systematic reviews. A series of emails with link to full reference, abstract and summary. Also, a visit from knowledge broker and access an on-line registry. |
| Williamson 2015(26) | 1) Policy influences: including media, public opinion, or stakeholder interests; 2) capacity, which refers to increasing the extent to which the organization and staff value research, increasing the extent to which the organization has the tools and systems needed to support research engagement and use, and increasing the extent to which staff have the skills and knowledge to engage with and use research; and 3) research engagement actions: which involve increasing access to research evidence, increasing skills to appraise research evidence, increasing the generation of new research or analyses by decision makers, and increasing the interaction between decision makers and researchers. |

References

1. Akl EA, Oxman AD, Herrin J, Vist GE, Terrenato I, Sperati F, et al. Framing of health information messages. Cochrane Database Syst Rev. 2011(12):CD006777.

2. ARMSTRONG R. Evidence-informed public health decision-making in local government 2011.

3. Ball S, Leach B, Bousfield J, Smith P, Marjanovic S. Arts-based approaches to public engagement with research: Lessons from a rapid review: RAND Corporation; 2021.

4. Bornbaum CC, Kornas K, Peirson L, Rosella LC. Exploring the function and effectiveness of knowledge brokers as facilitators of knowledge translation in health-related settings: a systematic review and thematic analysis. Implement Sci. 2015;10:162.

5. Brown A, Barnes C, Byaruhanga J, McLaughlin M, Hodder RK, Booth D, et al. Effectiveness of Technology-Enabled Knowledge Translation Strategies in Improving the Use of Research in Public Health: Systematic Review. J Med Internet Res. 2020;22(7):e17274.

6. Bunn F, Sworn K. Strategies to promote the impact of systematic reviews on healthcare policy: a systematic review of the literature. Evidence & Policy: A Journal of Research, Debate and Practice. 2011;7(4):403-28.

7. Chambers D, Wilson PM, Thompson CA, Hanbury A, Farley K, Light K. Maximizing the impact of systematic reviews in health care decision making: a systematic scoping review of knowledge-translation resources. Milbank Q. 2011;89(1):131-56.

8. Christine C, Susan C, Lisa D, Wendy G. What are the effects of interventions to improve the uptake of evidence from health research into policy in low and middle-income countries. Final report to DFID. 2011.

9. Dodd M, Ivers R, Zwi AB, Rahman A, Jagnoor J. Investigating the process of evidence-informed health policymaking in Bangladesh: a systematic review. Health Policy Plan. 2019;34(6):469-78.

10. Fadlallah R, El-Jardali F, Nomier M, Hemadi N, Arif K, Langlois EV, et al. Using narratives to impact health policy-making: A systematic review. Health Research Policy and Systems. 2019;17(1).

11. Haynes A, Rowbotham SJ, Redman S, Brennan S, Williamson A, Moore G. What can we learn from interventions that aim to increase policy-makers' capacity to use research? A realist scoping review. Federal Science Library - Canada. 2018;16(1).

12. LaRocca R, Yost J, Dobbins M, Ciliska D, Butt M. The effectiveness of knowledge translation strategies used in public health: a systematic review. BMC Public Health. 2012;12:751.

13. Mitton C, Adair CE, McKenzie E, Patten SB, Waye Perry B. Knowledge transfer and exchange: review and synthesis of the literature. Milbank Q. 2007;85(4):729-68.

14. Moore G, Redman S, Haines M, Todd A. What works to increase the use of research in population health policy and programmes: a review. Evidence & Policy: A Journal of Research, Debate and Practice. 2011;7(3):277-305.

15. Murthy L, Shepperd S, Clarke MJ, Garner SE, Lavis JN, Perrier L, et al. Interventions to improve the use of systematic reviews in decision-making by health system managers, policy makers and clinicians. Cochrane Database Syst Rev. 2012(9):Cd009401.

16. Partridge ACR, Mansilla C, Randhawa H, Lavis JN, El-Jardali F, Sewankambo NK. Lessons learned from descriptions and evaluations of knowledge translation platforms supporting evidence-informed policy-making in low- and middle-income countries: a systematic review. Health Res Policy Syst. 2020;18(1):127.

17. Perrier L, Mrklas K, Lavis JN, Straus SE. Interventions encouraging the use of systematic reviews by health policymakers and managers: a systematic review. Implement Sci. 2011;6:43.

18. Petkovic J, Welch V, Jacob MH, Yoganathan M, Ayala AP, Cunningham H, et al. The effectiveness of evidence summaries on health policymakers and health system managers use of evidence from systematic reviews: a systematic review. Implementation Science. 2016;11:1-14.

19. Quinn E, Huckel-Schneider C, Campbell D, Seale H, Milat AJ. How can knowledge exchange portals assist in knowledge management for evidence-informed decision making in public health? BMC public health. 2014;14:443.

20. Sarkies MN, Bowles KA, Skinner EH, Haas R, Lane H, Haines TP. The effectiveness of research implementation strategies for promoting evidence-informed policy and management decisions in healthcare: a systematic review. Implement Sci. 2017;12(1):132.

21. Tait H, Williamson A. A literature review of knowledge translation and partnership research training programs for health researchers. Health research policy and systems. 2019;17(1):1-14.

22. Tate K, Hewko S, McLane P, Baxter P, Perry K, Armijo-Olivo S, et al. Learning to lead: a review and synthesis of literature examining health care managers' use of knowledge. Journal of Health Services Research and Policy. 2019;24(1):57-70.

23. Uneke CJ, Sombie I, Johnson E, Uneke BI. Lessons Learned from Strategies for Promotion of Evidence-to-Policy Process in Health Interventions in the ECOWAS Region: A Rapid Review. Nigerian medical journal : journal of the Nigeria Medical Association. 2020;61(5):227-36.

24. Uneke CJ, Sombie I, Keita N, Lokossou V, Johnson E, Ongolo-Zogo P. An assessment of policymakers' engagement initiatives to promote evidence informed health policy making in Nigeria. The Pan African medical journal. 2017;27:57.

25. Wallace J, Byrne C, Clarke M. Improving the uptake of systematic reviews: a systematic review of intervention effectiveness and relevance. BMJ Open. 2014;4(10):e005834.

26. Williamson A, Makkar SR, McGrath C, Redman S. How Can the Use of Evidence in Mental Health Policy Be Increased? A Systematic Review. Psychiatric services (Washington, DC). 2015;66(8):appips201400329.

27. Campbell D, Moore G. Increasing the use of research in population health policies and programs: a rapid review. Public Health Research & Practice.
