# Supplementary material for: Assessing the impact of knowledge communication and dissemination strategies targeted at health policy-makers and managers: an overview of systematic reviews
Source: Health Res Policy Syst. 2021 Dec 6;19:140. doi: 10.1186/s12961-021-00780-4 (PMC8645346; doi:10.1186/s12961-021-00780-4)
Supplement: Supplementary file 7 — Additional file 7. Results and evidence statements. [file 12961_2021_780_MOESM7_ESM.docx]

**Additional file 7**

**Table 1. Types of outcomes measures**

| **Last name, year** | Awareness | Knowledge | Behavioural intentions | Evidence use | Making decisions | Understanding | Perceptions | Persuasiveness | Cost/Cost effectiveness | Attitudes/beliefs | Learning | Other |
| --- | --- | --- | --- | --- | --- | --- | --- | --- | --- | --- | --- | --- |
| Akl 2011(1) |  | X | X |  | X |  | X | X |  |  |  |  |
| Armstrong 2011(2) |  |  |  | X |  |  |  |  |  |  |  |  |
| Ball 2021(3) |  |  |  |  |  |  |  |  |  |  |  | Engagement |
| Bornbaum 2015 (4) |  | X |  |  | X |  |  |  |  |  |  | Skills |
| Brown 2020 (5) | X | X | X |  | X |  |  |  |  | X |  |  |
| Bunn 2011 (6) |  |  | X |  |  |  | X |  |  |  |  | Value on research evidence |
| Campbell 2018(7) | X | X |  |  | X | X |  |  |  |  |  | Partnerships and research co-production |
| Chambers 2011 (8) |  |  |  |  |  |  | X |  |  |  |  | Engagement |
| Christine(9) |  |  |  |  | X |  |  |  |  | X |  | Skills, scaling-up of intervention, health outcomes |
| Dodd 2019(10) | X | X |  | X |  |  |  | X | X |  | X | Policy change |
| Fadlallah 2019(11) | X | X |  |  | X |  |  |  |  |  |  |  |
| Haynes 2018(12) |  | X |  | X | X | X |  |  |  |  |  | Discussion about the evidence |
| LaRocca 2012 (13) |  | X |  | X | X |  |  |  |  |  |  |  |
| Mitton 2007 (14) |  |  | X | X | X | X |  | X |  |  | X | Self-reflection, policy development |
| Moore 2011(15) |  | X |  | X |  |  | X |  |  |  |  | Organisational research receptivity |
| Murthy 2012(16) | X |  |  | X | X |  |  |  |  |  |  | Acceptability |
| Partridge 2010(17) |  |  | X |  |  |  |  |  |  |  |  | Intention to act |
| Perrier 2011(18) |  |  |  | X | X | X |  |  |  |  |  |  |
| Petkovic 2016 (19) |  | X | X | X | X | X | X |  |  | X |  | Credibility |
| Quinn 2014(20) |  |  |  | X |  |  |  |  |  |  |  | Retaining users, research culture |
| Sarkies 2017(21) |  | X | X |  | X | X |  |  |  | X | X | Reaction |
| Tait 2019(22) |  | X |  | X |  | X |  |  |  |  | X | Discussion about the evidence |
| Tate 2019(23) |  | X |  | X |  | X |  |  |  |  |  |  |
| Uneke 2017(24) |  | X |  |  |  |  |  |  |  |  | X | Competences |
| Uneke 2020(25) |  |  |  |  |  | X |  |  |  |  | X | Sustainability of EIPM, Health outcome |
| Wallace 2014(26) |  |  |  |  | X |  |  |  |  |  |  |  |
| Williamson 2015(27) |  |  |  | X |  |  | X |  |  |  |  |  |

**Table 2. Studies by type of intervention and categories of evidence – Communication techniques**

| **Sufficient evidence** | **Some evidence** | **Insufficient evidence** | **Insufficient evidence to determine effectiveness** |
| --- | --- | --- | --- |
| **Tailoring the message** | | | |
| - | La Rocca 2012 | Bunn 2011  Moore 2011  Murthy 2012  Perrier 2011  Quinn 2014  Sarkies 2017  Wallace 2014 | Campbell 2018 |
| **Targeting the message** | | | |
| - | - | Armstrong 2011  Bunn 2011  Moore 2011  Perrier 2011  Sarkies 2017 | Campbell 2018 |
| **Using narratives** | | | |
| - | - | Ball 2021 | Fadallah 2019 |
| Framing the message | | | |
| - | Akl 2011 (indirect evidence) | - | Campbell 2019  Dood 2019 |
| **Using different presentation formats** | | | |
| - | Petkovic 2016 | - | - |
| **Multicomponent communication techniques** | | | |
| - |  | Ball 2021  Williamson 2015 | - |

**Table 3. Studies by type of intervention and categories of evidence – Dissemination strategies**

| **Sufficient evidence** | **Some evidence** | **Insufficient evidence** | **Insufficient evidence to determine effectiveness** |
| --- | --- | --- | --- |
| **Increase reach** | | | |
| - | La Rocca 2012 | Chambers 2011  Murthy 2012  Perrier 2011  Quinn 2014  Tait 2019  Wallace 2014  Williamson 2015 | Campbell 2018  Dood 2019 |
| **Increase people motivation** | | | |
| - | La Rocca 2012 | Armstrong 2011  Bornbaum 2015  Bunn 2011  Moore 2011  Murthy 2012  Perrier 2011  Williamson 2015 | Campbell 2018  Christine 2011  Dood 2019  Mitton 2007 |
| **Increase people ability to use** | | | |
| - | Haynes 2018 | Ball 2021  Moore 2011  Tait 2019  Williamson 2015 | Campbell 2018  Christine 2011  Dood 2019  Tate 2019  Uneke 2017 |
| **Multifaceted dissemination strategy** | | | |
| - | Brown 2020  LaRocca 2012 | Williamson 2015 | Partridge 2020  Uneke 2020 |

**References**

1. Akl EA, Oxman AD, Herrin J, Vist GE, Terrenato I, Sperati F, et al. Framing of health information messages. Cochrane Database Syst Rev. 2011(12):CD006777.

2. ARMSTRONG R. Evidence-informed public health decision-making in local government 2011.

3. Ball S, Leach B, Bousfield J, Smith P, Marjanovic S. Arts-based approaches to public engagement with research: Lessons from a rapid review: RAND Corporation; 2021.

4. Bornbaum CC, Kornas K, Peirson L, Rosella LC. Exploring the function and effectiveness of knowledge brokers as facilitators of knowledge translation in health-related settings: a systematic review and thematic analysis. Implement Sci. 2015;10:162.

5. Brown A, Barnes C, Byaruhanga J, McLaughlin M, Hodder RK, Booth D, et al. Effectiveness of Technology-Enabled Knowledge Translation Strategies in Improving the Use of Research in Public Health: Systematic Review. J Med Internet Res. 2020;22(7):e17274.

6. Bunn F, Sworn K. Strategies to promote the impact of systematic reviews on healthcare policy: a systematic review of the literature. Evidence & Policy: A Journal of Research, Debate and Practice. 2011;7(4):403-28.

7. Campbell D, Moore G. Increasing the use of research in population health policies and programs: a rapid review. Public Health Research & Practice.

8. Chambers D, Wilson PM, Thompson CA, Hanbury A, Farley K, Light K. Maximizing the impact of systematic reviews in health care decision making: a systematic scoping review of knowledge-translation resources. Milbank Q. 2011;89(1):131-56.

9. Christine C, Susan C, Lisa D, Wendy G. What are the effects of interventions to improve the uptake of evidence from health research into policy in low and middle-income countries. Final report to DFID. 2011.

10. Dodd M, Ivers R, Zwi AB, Rahman A, Jagnoor J. Investigating the process of evidence-informed health policymaking in Bangladesh: a systematic review. Health Policy Plan. 2019;34(6):469-78.

11. Fadlallah R, El-Jardali F, Nomier M, Hemadi N, Arif K, Langlois EV, et al. Using narratives to impact health policy-making: A systematic review. Health Research Policy and Systems. 2019;17(1).

12. Haynes A, Rowbotham SJ, Redman S, Brennan S, Williamson A, Moore G. What can we learn from interventions that aim to increase policy-makers' capacity to use research? A realist scoping review. Federal Science Library - Canada. 2018;16(1).

13. LaRocca R, Yost J, Dobbins M, Ciliska D, Butt M. The effectiveness of knowledge translation strategies used in public health: a systematic review. BMC Public Health. 2012;12:751.

14. Mitton C, Adair CE, McKenzie E, Patten SB, Waye Perry B. Knowledge transfer and exchange: review and synthesis of the literature. Milbank Q. 2007;85(4):729-68.

15. Moore G, Redman S, Haines M, Todd A. What works to increase the use of research in population health policy and programmes: a review. Evidence & Policy: A Journal of Research, Debate and Practice. 2011;7(3):277-305.

16. Murthy L, Shepperd S, Clarke MJ, Garner SE, Lavis JN, Perrier L, et al. Interventions to improve the use of systematic reviews in decision-making by health system managers, policy makers and clinicians. Cochrane Database Syst Rev. 2012(9):Cd009401.

17. Partridge ACR, Mansilla C, Randhawa H, Lavis JN, El-Jardali F, Sewankambo NK. Lessons learned from descriptions and evaluations of knowledge translation platforms supporting evidence-informed policy-making in low- and middle-income countries: a systematic review. Health Res Policy Syst. 2020;18(1):127.

18. Perrier L, Mrklas K, Lavis JN, Straus SE. Interventions encouraging the use of systematic reviews by health policymakers and managers: a systematic review. Implement Sci. 2011;6:43.

19. Petkovic J, Welch V, Jacob MH, Yoganathan M, Ayala AP, Cunningham H, et al. The effectiveness of evidence summaries on health policymakers and health system managers use of evidence from systematic reviews: a systematic review. Implementation Science. 2016;11:1-14.

20. Quinn E, Huckel-Schneider C, Campbell D, Seale H, Milat AJ. How can knowledge exchange portals assist in knowledge management for evidence-informed decision making in public health? BMC public health. 2014;14:443.

21. Sarkies MN, Bowles KA, Skinner EH, Haas R, Lane H, Haines TP. The effectiveness of research implementation strategies for promoting evidence-informed policy and management decisions in healthcare: a systematic review. Implement Sci. 2017;12(1):132.

22. Tait H, Williamson A. A literature review of knowledge translation and partnership research training programs for health researchers. Health research policy and systems. 2019;17(1):1-14.

23. Tate K, Hewko S, McLane P, Baxter P, Perry K, Armijo-Olivo S, et al. Learning to lead: a review and synthesis of literature examining health care managers' use of knowledge. Journal of Health Services Research and Policy. 2019;24(1):57-70.

24. Uneke CJ, Sombie I, Keita N, Lokossou V, Johnson E, Ongolo-Zogo P. An assessment of policymakers' engagement initiatives to promote evidence informed health policy making in Nigeria. The Pan African medical journal. 2017;27:57.

25. Uneke CJ, Sombie I, Johnson E, Uneke BI. Lessons Learned from Strategies for Promotion of Evidence-to-Policy Process in Health Interventions in the ECOWAS Region: A Rapid Review. Nigerian medical journal : journal of the Nigeria Medical Association. 2020;61(5):227-36.

26. Wallace J, Byrne C, Clarke M. Improving the uptake of systematic reviews: a systematic review of intervention effectiveness and relevance. BMJ Open. 2014;4(10):e005834.

27. Williamson A, Makkar SR, McGrath C, Redman S. How Can the Use of Evidence in Mental Health Policy Be Increased? A Systematic Review. Psychiatric services (Washington, DC). 2015;66(8):appips201400329.
