# Supplementary material for: Assessing the impact of knowledge communication and dissemination strategies targeted at health policy-makers and managers: an overview of systematic reviews
Source: Health Res Policy Syst. 2021 Dec 6;19:140. doi: 10.1186/s12961-021-00780-4 (PMC8645346; doi:10.1186/s12961-021-00780-4)
Supplement: Supplementary file 8 — Additional file 8. Frameworks/theories/models from included SRs. [file 12961_2021_780_MOESM8_ESM.docx]

Frameworks/theories/models from included SRs

| Author | Framework |
| --- | --- |
| Akl 2011 | Rothman and Salovey built on Kahneman and Tversky’s work to extend these concepts to the health domain. They hypothesized that gain frames would be more effective for disease prevention and recuperative (therapeutic) behaviors and that loss-frames would be more effective for disease detection (screening) behaviors |
| Ball 2021 | Arts-based Knowledge Translation (ABKT) Framework – adapted and abridged from Kukkonen and Cooper (2017; 2018), and the Guiding Arts-Based Research Assessment framework (GABRA), was developed specifically to assess the quality and effectiveness of arts-based works used to represent research findings |
| Bornbaum 2015 | KB domains previously described by other authors (Ward and Oldham & McLean) |
| Dodd 2019 | Policy framework designed by Shiffman and Smith |
| Fadlallah 2019 | Elaboration likelihood model (ELM), Green’s transportation imagery model, Entertainment overcoming resistance model, Kahneman’s two-system way of thinking |
| Haynes 2018 | Multiple. Include an inductive approach to analysis guided by realist thinking rather than starting with an a priori framework or conceptual categories. |
| Mitton 2007 | Five frameworks to guide KTE initiatives (Dobbins 2002, Hanney 2003, Ebener 2006, Lavis 2003, Jacobson, Butterill and Goering 2003) |
| Quinn 2014 | Authors used the domains of knowledge management as defined by Lee et Al. 2010 |
| Sarkies 2017 | Kirkpatrick’s Evaluation Model Hierarchy |
| Tait 2019 | Theory of KT. Synthesis: The program reported by Gerrish and Piercy [[30](https://health-policy-systems.biomedcentral.com/articles/10.1186/s12961-019-0497-z#ref-CR30)] aimed to build skills in applying KT frameworks in practise, evidence appraisal, skills to facilitate practice change and evaluation. Jones et al. [[31](https://health-policy-systems.biomedcentral.com/articles/10.1186/s12961-019-0497-z#ref-CR31)], Kho et al. [[36](https://health-policy-systems.biomedcentral.com/articles/10.1186/s12961-019-0497-z#ref-CR36)] and Uneke et al. [[35](https://health-policy-systems.biomedcentral.com/articles/10.1186/s12961-019-0497-z#ref-CR35)] all focused on theory of KT and planning for KT. Santacroce et al. [[32](https://health-policy-systems.biomedcentral.com/articles/10.1186/s12961-019-0497-z#ref-CR32)] proposed a framework of six research translation themes (dissemination oriented) embedded into an existing research curriculum. The workshop [[33](https://health-policy-systems.biomedcentral.com/articles/10.1186/s12961-019-0497-z#ref-CR33)] and secondment program [[34](https://health-policy-systems.biomedcentral.com/articles/10.1186/s12961-019-0497-z#ref-CR34)] described by Uneke et al. aimed to facilitate networking between researchers and policy-makers and to build capacity for evidenced-based policy work. The session reports by Leung et al. [[37](https://health-policy-systems.biomedcentral.com/articles/10.1186/s12961-019-0497-z#ref-CR37)] and Bhogal et al. [[38](https://health-policy-systems.biomedcentral.com/articles/10.1186/s12961-019-0497-z#ref-CR38)] described the aim as being to increase participant skills and confidence in developing KT strategies. |
| Wallace 2014 | A framework for integrating different types of evidence in systematic reviews for public policy. (Oliver S, Harden A, Rees R, et al. An emerging framework for including different types of evidence in systematic reviews for public policy. Evaluation 2005;11:428–46.) |
| Williamson 2015 | SPIRIT (Supporting Policy In health with Research: an Intervention Trial) action framework |

References

1. Akl EA, Oxman AD, Herrin J, Vist GE, Terrenato I, Sperati F, et al. Framing of health information messages. Cochrane Database Syst Rev. 2011(12):CD006777.

2. Bornbaum CC, Kornas K, Peirson L, Rosella LC. Exploring the function and effectiveness of knowledge brokers as facilitators of knowledge translation in health-related settings: a systematic review and thematic analysis. Implement Sci. 2015;10:162.

3. Dodd M, Ivers R, Zwi AB, Rahman A, Jagnoor J. Investigating the process of evidence-informed health policymaking in Bangladesh: a systematic review. Health Policy Plan. 2019;34(6):469-78.

4. Fadlallah R, El-Jardali F, Nomier M, Hemadi N, Arif K, Langlois EV, et al. Using narratives to impact health policy-making: A systematic review. Health Research Policy and Systems. 2019;17(1).

5. Haynes A, Rowbotham SJ, Redman S, Brennan S, Williamson A, Moore G. What can we learn from interventions that aim to increase policy-makers' capacity to use research? A realist scoping review. Federal Science Library - Canada. 2018;16(1).

6. Mitton C, Adair CE, McKenzie E, Patten SB, Waye Perry B. Knowledge transfer and exchange: review and synthesis of the literature. Milbank Q. 2007;85(4):729-68.

7. Quinn E, Huckel-Schneider C, Campbell D, Seale H, Milat AJ. How can knowledge exchange portals assist in knowledge management for evidence-informed decision making in public health? BMC public health. 2014;14:443.

8. Sarkies MN, Bowles KA, Skinner EH, Haas R, Lane H, Haines TP. The effectiveness of research implementation strategies for promoting evidence-informed policy and management decisions in healthcare: a systematic review. Implement Sci. 2017;12(1):132.

9. Tait H, Williamson A. A literature review of knowledge translation and partnership research training programs for health researchers. Health research policy and systems. 2019;17(1):1-14.

10. Wallace J, Byrne C, Clarke M. Improving the uptake of systematic reviews: a systematic review of intervention effectiveness and relevance. BMJ Open. 2014;4(10):e005834.

11. Ball S, Leach B, Bousfield J, Smith P, Marjanovic S. Arts-based approaches to public engagement with research: Lessons from a rapid review: RAND Corporation; 2021.

12. Williamson A, Makkar SR, McGrath C, Redman S. How can the use of evidence in mental health policy be increased? A systematic review. Psychiatric Services. 2015;66(8):783-97.
